# From mechanism to substratome: Unraveling mysteries of γ-secretase

**DOI:** 10.1016/j.jbc.2026.113287

**Published:** 2026-06-23

**Authors:** Harald Steiner, Stephan Breimann, Frits Kamp, Dmitrij Frishman

**Affiliations:** 1 Division of Metabolic Biochemistry, Biomedical Center (BMC), LMU Munich, München, Germany; 2 German Center for Neurodegenerative Diseases (DZNE), DZNE Munich, München, Germany; 3 Department of Bioinformatics, Technical University of Munich, Freising, Germany

**Keywords:** Alzheimer’s disease, amyloid-beta (Aβ), amyloid precursor proein (APP), gamma-secretase, gamma-secretase modulator (GSM), intramembrane proteolysis, presenilin

## Abstract

γ-Secretase is a pivotal membrane-embedded protease that cleaves more than 150 single-span membrane proteins within their transmembrane domains. While γ-secretase is involved in a wide range of physiological processes, it is best known for its critical role in Alzheimer’s disease, where it cleaves a C-terminal fragment of the amyloid precursor protein into small aggregation-prone and neurotoxic peptides. However, how γ-secretase recognizes and recruits its substrates, how it binds and unfolds them, where drug-binding sites are located, and what the full range of its substrates and functions is, have all remained unknown. These long-standing questions have been at the forefront of research for the past decade and are now increasingly being solved. In this review, we outline how recent advances in structural biology, biochemistry, and computational biology have helped to elucidate these mysteries. We also highlight future research directions needed to achieve a comprehensive understanding of this fascinating enzyme, a major therapeutic target for which Alzheimer’s disease drugs are now on the horizon.

The regulated breakdown of proteins by proteolytic cleavage is a fundamental process essential to life (1). Proteases, the enzymes that catalyze this process by cleaving peptide bonds in their substrates, occur in both soluble and membrane-bound forms. Intramembrane proteases constitute a subgroup of membrane-bound proteases that carry their active site residues in adjacent transmembrane helices (2, 3). This unusual location allows them to cleave substrate proteins directly within their transmembrane domain (TMD). Intramembrane proteases include the site-2 metalloproteases, rhomboid serine proteases, the glutamate protease Ras and α-factor converting enzyme 1, as well as presenilin, signal peptide peptidase (SPP), and related proteases that belong to the aspartate protease family (2, 3).

## γ-Secretase is an intramembrane protease complex with a sophisticated cleavage mechanism

Unlike all other intramembrane proteases, presenilin associates with three additional proteins to form the protease complex known as γ-secretase (4, 5, 6). In humans, this tetrameric complex consists of presenilin 1 or 2 (PS1, PS2) as the catalytic subunit, nicastrin (NCT), anterior pharynx defective 1a or 1b (APH-1a, APH-1b) and presenilin enhancer 2 (PEN-2). γ-Secretase is critically implicated in Alzheimer’s disease (AD), the most common form of dementia, through its role in the proteolytic processing of the amyloid precursor protein (APP) into amyloid-β peptide (Aβ) (7). Aβ aggregates in the brain of affected individuals and triggers the disease (8). Consistent with the widely held view that Aβ is the root cause of AD, its removal by brain-penetrating monoclonal antibodies has recently shown clinical benefit in phase 3 trials, leading to the approval of these molecules as the first disease-modifying AD therapeutics (9). Given its essential role in Aβ generation, γ-secretase has been intensely studied for nearly 3 decades as a major AD drug target (10). Along the way, it became clear that γ-secretase has an ever-growing list of more than 150 substrates and a broad functional spectrum (11, 12). These include the Notch receptor NOTCH1, a major γ-secretase substrate that regulates cell differentiation throughout life, as well as many other substrates involved in physiologically important processes. Increased processing of NOTCH1 by γ-secretase can lead to cancers such as T-cell acute lymphoblastic leukemia (T-ALL) (13).

### Substrate cleavage pathways of γ-secretase

The majority of γ-secretase substrates are single-span type I membrane proteins, i.e., with an extracellular N-terminus. The proteolytic attack by γ-secretase occurs after an initial cleavage by sheddases—proteases that remove most of the substrate ectodomain, leaving a C-terminal fragment (CTF) in the membrane (14)—a process referred to as ectodomain shedding. Major sheddases are the α-secretases ADAM10 and ADAM17 (15), and the β-secretase BACE1 (16). In the case of APP, these secretases generate the large soluble ectodomains APPsα and APPsβ.

**Figure 1:**
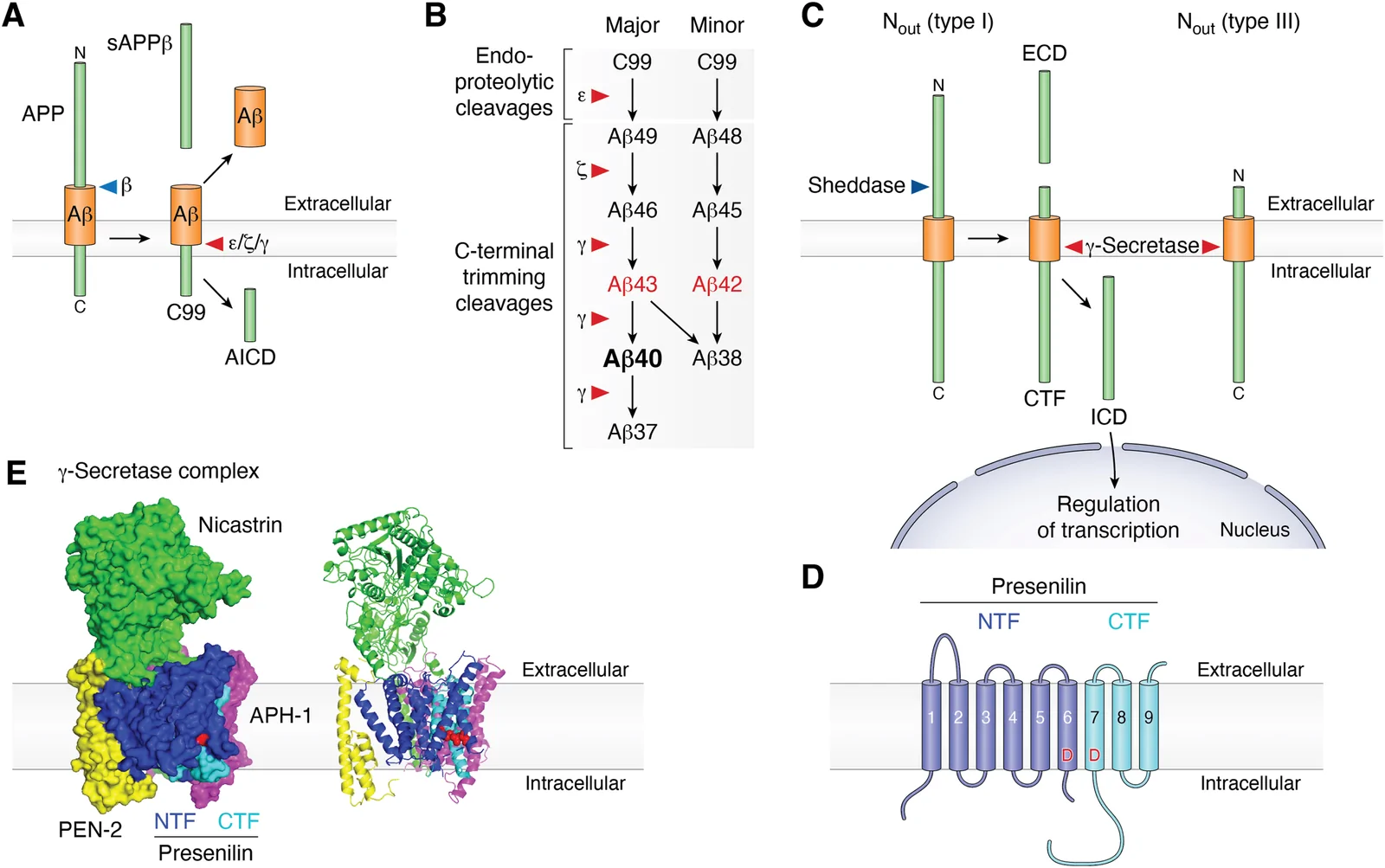
**Intramembrane proteolysis by γ-secretase.***A*, Amyloidogenic processing pathway of APP. Greek symbols indicate the APP cleavages at the β-site by β-secretase, releasing the soluble APP ectodomain (sAPPβ), and the sequential γ-secretase cleavages at the ε-, ζ-, and γ-sites, liberating the Aβ and AICD cleavage products. *B*, sequential cleavages of C99 into Aβ species by γ-secretase. Greek symbols indicate the γ-secretase cleavages at the ε-, ζ-, and γ-sites by which the indicated Aβ species are generated in a major and minor product line. The major peptide Aβ40 is highlighted in bold; the pathogenic species Aβ42 and Aβ43 are depicted in *red*. *C*, γ-Secretase substrate types. Established substrates comprise single-span membrane proteins with an extracellular N-terminus (N_out_) of type I or type III (*i.e.*, synthesized with or without a signal sequence, respectively). Type I membrane protein substrates require a sheddase to remove a large portion of their extracellular domain (ECD), generating a CTF that is cleaved next by γ-secretase, whereas type III membrane protein substrates with naturally short ectodomains are cleaved by γ-secretase as full-length proteins. After γ-secretase cleavage, some substrate ICDs translocate to the nucleus and function in transcription regulation. *D*, transmembrane topology of the γ-secretase catalytic subunit presenilin. NTF and CTF are highlighted in blue and cyan, respectively. Active site aspartate residues are depicted in one-letter code in *red*. *E*, cryo-EM structure of the γ-secretase complex (PDB 5FN3) in surface (*left panel)* and cartoon (*right panel*) representation. Presenilin NTF and CTF are colored as in (*D*), NCT is shown in *green*, PEN-2 in yellow and APH-1 in *magenta*. Active site aspartate residues are depicted as *red spheres*.

As exemplified by the well-studied processing of APP into Aβ, γ-secretase cleaves within the TMD of C99, the 99 amino acid APP CTF generated by the initial cleavage of APP by β-secretase (Fig. 1*A*). C99 cleavage occurs at the C-terminal part of its TMD at the ε-site through the endoproteolytic activity of γ-secretase and releases the intracellular domain (ICD) of APP, the AICD, into the cytosol. The ε-site cleavage is heterogeneous and occurs at two positions thereby generating Aβ49 and Aβ48 (17, 18, 19, 20). These long Aβ forms are subsequently cleaved in a stepwise manner by the carboxy-peptidase-like activity of γ-secretase (21, 22, 23) (Fig. 1*B*). In the main pathway, Aβ49 is cleaved *via* the intermediate peptides Aβ46 and Aβ43 to Aβ40, the predominant Aβ product. In a parallel minor pathway, Aβ48 is converted to Aβ38 *via* Aβ45 and Aβ42 as intermediates. These sequential trimming steps result in the release of the corresponding small byproducts, which are mostly tripeptides. Both Aβ42 and Aβ43 are constituents of pathogenic Aβ aggregates deposited in the AD brain as amyloid plaques, which, in their mature form, also contain Aβ40 (24, 25). The two principal pathways can be switched at several steps, particularly by the conversion of Aβ43 to Aβ38 (26, 27). It is likely that γ-secretase processes other substrates sequentially as well, as Aβ-like cleavage products and ICDs arising from distinct TMD cleavage sites have also been identified for NOTCH1 and a handful of other substrates (28, 29, 30, 31). While a principal role of γ-secretase is the clearing of CTFs remaining in the membrane after ectodomain shedding of single-span type I membrane proteins (32), γ-secretase is often also critically involved in signaling. As was first demonstrated for NOTCH1 (33), in many cases, substrate ICDs can translocate to the nucleus and regulate gene transcription (12) (Fig. 1*C*).

A small subset of γ-secretase substrates is cleaved as full-length proteins. These proteins are type III membrane proteins with naturally short N-terminal ectodomains of fewer than 75 residues (34, 35) (Fig. 1*C*). There have also been reports indicating that γ-secretase may cleave polytopic membrane proteins (36, 37, 38). The evidence for direct cleavage by γ-secretase is still limited, and the mechanisms underlying substrate recognition and cleavage of such proteins have not yet been elucidated. However, cleavage of polytopic membrane proteins by intramembrane proteases is not unprecedented. It has previously been shown for rhomboid proteases (39, 40, 41, 42, 43, 44) and may therefore also apply to other intramembrane proteases, including γ-secretase.

### Physiological functions of the γ-secretase cleavage products of APP

While ICD-mediated signaling had been firmly established as NOTCH1 function, the question of whether the AICD may perform a similar nuclear signaling function has remained controversial. Notably, since the initial observation that AICD can engage in the formation of a transcriptionally active protein complex as demonstrated by reporter gene assays (45), subsequently identified potential AICD-regulated target genes could not be confirmed at the endogenous protein (46) or transcriptional levels (47). In fact, as suggested by *in vivo* studies of APP knockout mice (48), APPsα may be the more functionally relevant APP domain.

Given its critical implication in AD pathogenesis and its role as the major therapeutic target, there has been considerable research activity regarding a potential physiological function(s) of Aβ in the brain, as this would have obvious implications for Aβ-lowering therapeutic approaches. These efforts have identified several potential physiological roles of Aβ, with its involvement in synaptic function and memory being among the better studied (49), given the long-known correlation between synapse loss and cognitive decline in AD (50, 51). As a main finding, it could be shown that Aβ is required for synaptic plasticity and memory formation at low physiological (picomolar) concentrations, by agonistically acting on α7 nicotinic acetylcholine receptors (52). Conversely, elevated (nanomolar) concentrations have been observed to disrupt these functions (52), consistent with the well-established Aβ-mediated synaptotoxicity under pathological conditions (53). The beneficial effects of Aβ are likely attributable to its N-terminal region as shown by the synaptic plasticity-enhancing activity of shorter Aβ peptides such as Aβ1-15 (54) and Aβ1-16 (55), which can be naturally produced in the brain by the combined action of β- and α-secretase (56, 57). However, the N-terminal Aβ1-16 sequence is also present at the C-terminal end of APPsα (termed CTα16 domain), which was shown to mediate the memory-enhancing synaptic functions of APP on the α7 nicotinic acetylcholine receptors (55, 58). Both molecules, Aβ and APPsα, therefore appear to promote synaptic plasticity and memory formation by virtue of this overlapping sequence. While increasing APPsα levels thus has therapeutic potential in the treatment of AD (59), which was recently further substantiated for CTα16 (60), levels of Aβ should probably not be lowered too much in anti-amyloid therapies.

### Presenilin is the catalytic heart of γ-secretase

Presenilin (Fig. 1*D*) is the central subunit of γ-secretase. Its catalytically active aspartate residues are located in TMDs 6 and 7 and face each other. Within the γ-secretase complex, presenilin is present as an N-terminal fragment (NTF) containing TMDs 1 to 6 and a CTF containing TMDs 7 to 9, which are generated by stepwise autoproteolytic cleavage within the large cytoplasmic loop (5, 61, 62, 63, 64, 65, 66). The identity of presenilin as a protease harboring the γ-secretase active site was not initially clear. Although the knockout of PS1 in mice caused a phenotype consistent with γ-secretase deficiency (*i.e.*, reduced Aβ levels accompanied by an accumulation of APP CTFs) (67, 68), the lack of similarity of presenilin to any other known protease together with the observation that overexpression of presenilin did not increase γ-secretase activity (*i.e.*, increased Aβ generation) (69) appeared incompatible with the hypothesis that it is identical with γ-secretase. This link was ultimately established by a series of further seminal observations. Pharmacological inhibition experiments suggested that γ-secretase belongs to the aspartate protease family (*e.g.*, using pepstatin and γ-secretase inhibitor (GSI) compounds designed to mimic the transition state of aspartate proteases) (70, 71, 72, 73). Indeed, presenilin was found to contain two conserved aspartates whose mutation inactivates γ-secretase (63, 64, 74). Moreover, presenilin could be affinity-labeled by GSIs (75, 76, 77), and also homology of a small sequence part including the TMD7 aspartate with an unrelated bacterial aspartate protease was identified (78). This signature GxGD (x, any amino acid) protease active site motif was subsequently found in all other aspartate intramembrane proteases (79, 80, 81).

Subsequent biochemical studies showed that, within the γ-secretase complex, presenilin is in direct contact with PEN-2, APH-1, and NCT (82, 83). Seminal cryo-electron microscopy (cryo-EM) studies later corroborated this subunit arrangement (84, 85, 86) (Fig. 1*E*). NCT is a single-span type I membrane protein with a large, highly glycosylated ectodomain, which covers the presenilin heterodimer. APH-1 is a polytopic membrane protein with seven TMDs and acts as a scaffold for presenilin and NCT within the complex. PEN-2 is the smallest subunit consisting of a single TMD and two membrane-penetrating segments forming a hairpin. PEN-2 is located in close proximity to NCT and presenilin and triggers the autoproteolysis of presenilin (4, 87). The γ-secretase complex is assembled stepwise in the early secretory pathway, forming a full complex containing presenilin maturated into NTF and CTF shortly after exit of the ER (88, 89, 90, 91). Following assembly and maturation, PS1-containing γ-secretases reach the plasma membrane as their final localization, while PS2-containing γ-secretases are directed to the endosomal/lysosomal system (92, 93).

### Familial AD mutations in γ-secretase change the composition of Aβ

**Figure 2:**
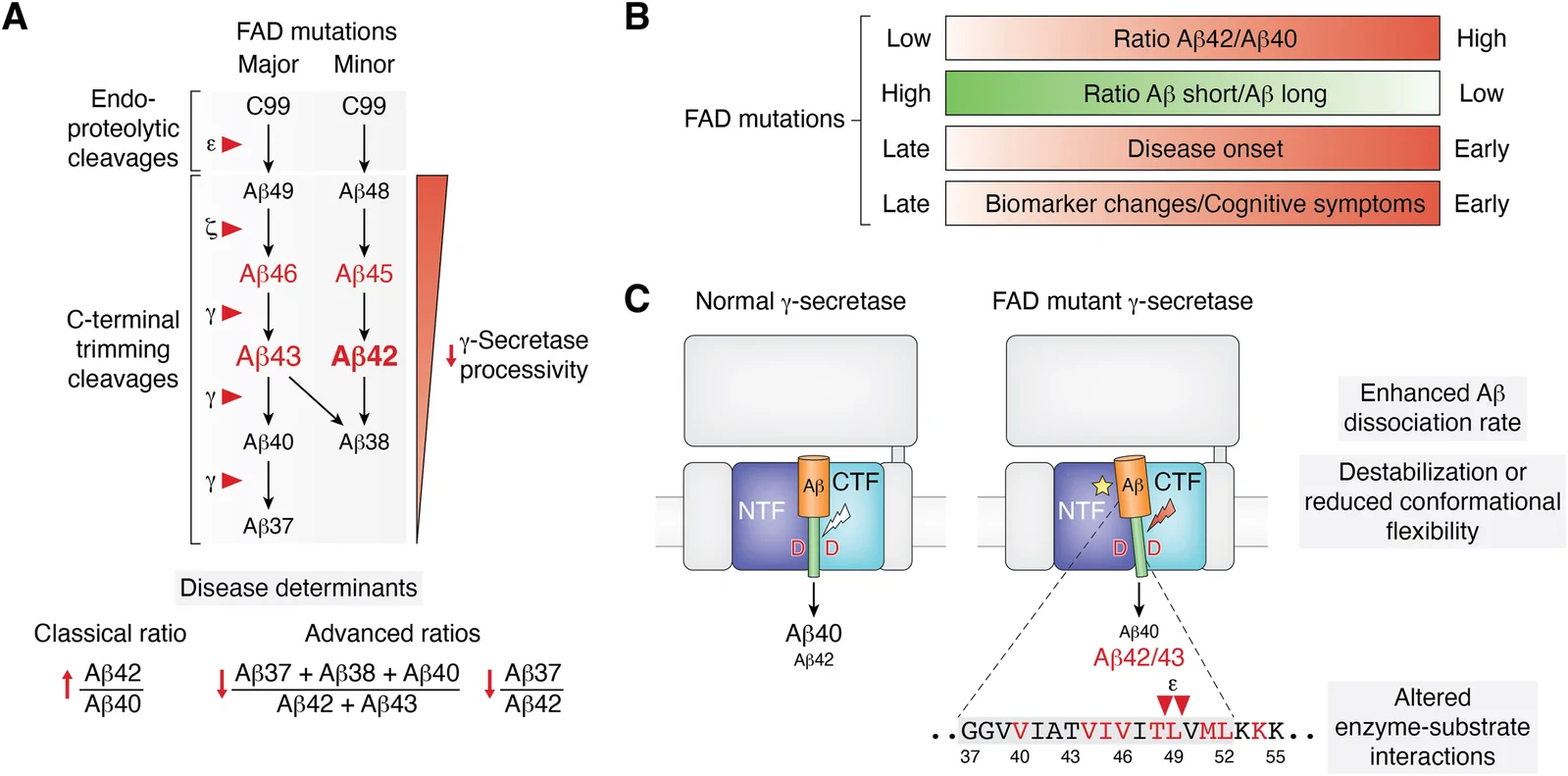
**Aβ generation by FAD mutants.***A*, FAD mutations in presenilin and in the γ-secretase cleavage site region of APP cause a reduced processivity of γ-secretase, leading to the accumulation of longer Aβ forms (*red*), mostly Aβ42 (highlighted in bold). Altered ratios of Aβ42 to Aβ40 or of short to long Aβ species, respectively, are determinants for AD. *B*, correlation of the ratio of Aβ42 to Aβ40 or, respectively, the ratio of short to long Aβ species generated by FAD mutations with disease onset and, as shown for PS1 FAD mutants, progression. *C*, presenilin FAD mutations display altered enzyme-substrate interactions. Residues in the respective part of the APP TMD (*gray*) and adjacent JMD that interact differently with FAD mutant γ-secretase are highlighted in *red*. Numbering of residues according to Aβ/C99 sequence. FAD mutant γ-secretase E–S complexes display enhanced dissociation rates for Aβ42/43. E–S complex destabilization has been suggested as a cause, but reduced conformational flexibility, *i.e.*, increased stability of the E–S complex, has also been proposed.

While AD typically begins after the age of 65, about 1% of the AD cases are rare, genetically inherited forms that can manifest as early as adolescence. These familial AD (FAD) forms are, in the vast majority of cases, due to mutations in PS1. The Alzheimer Research Forum website (Alzforum; www.alzforum.org) (94) classifies more than 200 PS1 FAD mutations as pathogenic/likely pathogenic in its mutations database (95). The mutations are distributed throughout presenilin, but cluster in its TMDs and cause a change in the profile of Aβ species produced by γ-secretase. As a consequence of the reduced processivity imposed by the FAD mutations (26, 96), this results in a relative increase of secreted Aβ42 (69, 97) and/or, but less frequently, Aβ43 (98, 99, 100) in the total pool of secreted Aβ species (Fig. 2*A*). These changes are also observed for PS2 FAD mutations, although these are much less prevalent than those in PS1 (69, 97, 101, 102). A few FAD mutations that cause this biochemical phenotype of changes in the relative production of longer Aβ species are also found in the APP TMD, where they localize to the region cleaved by γ-secretase (97, 102).

In contrast to Aβ37-43, very long Aβ forms (≥Aβ45) are not secreted and may cause toxicity if they remain unprocessed in the membrane, potentially contributing to FAD pathogenesis. Increased generation of very long Aβ was observed in cell-free assays for several presenilin FAD mutants (103). Similarly, APP TMD FAD mutations have also been found to cause an inefficient degradation of very long Aβ species by γ-secretase in such assays (104). However, the disease relevance of these observations remains unclear, as reduced γ-secretase activity is often observed in such assays, presumably due to enzyme destabilization following detergent solubilization (105). Nevertheless, interestingly, some evidence for the accumulation of very long Aβ in the brain has been reported in a patient carrying the PS1 E280A mutation (106).

An increased ratio of secreted Aβ42 over Aβ40 has been linked with an earlier disease onset (107, 108) (Fig. 2, *A* and *B*). The correlation with disease onset based on the classical, commonly used Aβ42/Aβ40 ratio can be refined by including the short species Aβ37 and Aβ38 as well as the longer Aβ43, in a ratio of Aβ37 + Aβ38 + Aβ40 over Aβ42 + Aβ43, thereby even allowing predictions of the disease onset in mutation carriers (101, 102, 109) or, alternatively, by using the Aβ37/ Aβ42 ratio (108). In addition, variations in γ-secretase activity affecting the generation of short Aβ species observed for more than 50 different PS1 FAD mutants correlated strongly with clinical, cognitive, and *in vivo* biomarker measures of the corresponding mutation carriers of the Dominantly Inherited Alzheimer’s Disease Network (DIAN) cohort enrolled in an observational study (109). These data will be useful for predicting disease onset and progression of newly discovered pathogenic PS1 variants as well as for variants with no clinical history or of unknown clinical significance.

### Molecular mechanisms of presenilin FAD mutants

The increased production of Aβ42 and Aβ43 by presenilin FAD mutations is thought to result from altered enzyme–substrate (E–S) complex interactions (Fig. 2*C*). This has been demonstrated by photoaffinity-labeling experiments with APP CTF substrates, which showed altered interactions in their cleavage site domain with presenilin FAD mutants (110, 111). Evidence for an altered E–S complex was also obtained by previous experiments showing that presenilin FAD mutations increase the dissociation rate of Aβ42 from the complex (26). Incomplete processing of Aβ42 to Aβ38 and of Aβ43 to Aβ40 could thus be understood as a consequence of the reduced residence time of the longer Aβ42/43 species at the γ-secretase active site. Consistent with these studies, it was found in cleavage assays measuring the temperature-dependence of γ-secretase activity that presenilin FAD mutations, as well as FAD mutations in the APP TMD, destabilize the E–S complex, causing a faster release of long Aβ species (105). The destabilizing effects of these FAD mutations, both in the free protein and within the E–S complex, were also predicted by several computational methods for calculating folding stability (112). However, although destabilization of the E–S complex can readily explain the abnormal production of pathogenic Aβ species by FAD mutants, more recent data challenge this mechanism and instead suggest that FAD mutations stabilize the E–S complex (113, 114). In this competing scenario, reduced conformational flexibility of the substrate in the active site of presenilin FAD mutant E–S complexes are thought to result in less efficient substrate processing and premature release of long Aβ species. Such stalling of E–S complexes has been suggested to underlie the synaptic degeneration observed in a *C. elegans* neurodegeneration model of FAD (113, 114). It is noteworthy that these toxic effects were also observed for mutant APP substrates that cannot be processed to Aβ42 and Aβ43. It is thus possible that stalled E–S complexes contribute to FAD pathogenesis independently of toxic Aβ aggregates. Whether stalled E–S complexes could even serve as a potential alternative trigger of AD pathogenesis remains an open question, as has been recently discussed (115). More research is needed to support this new hypothesis.

### γ-Secretase substrate recruitment involves exosites

**Figure 3:**
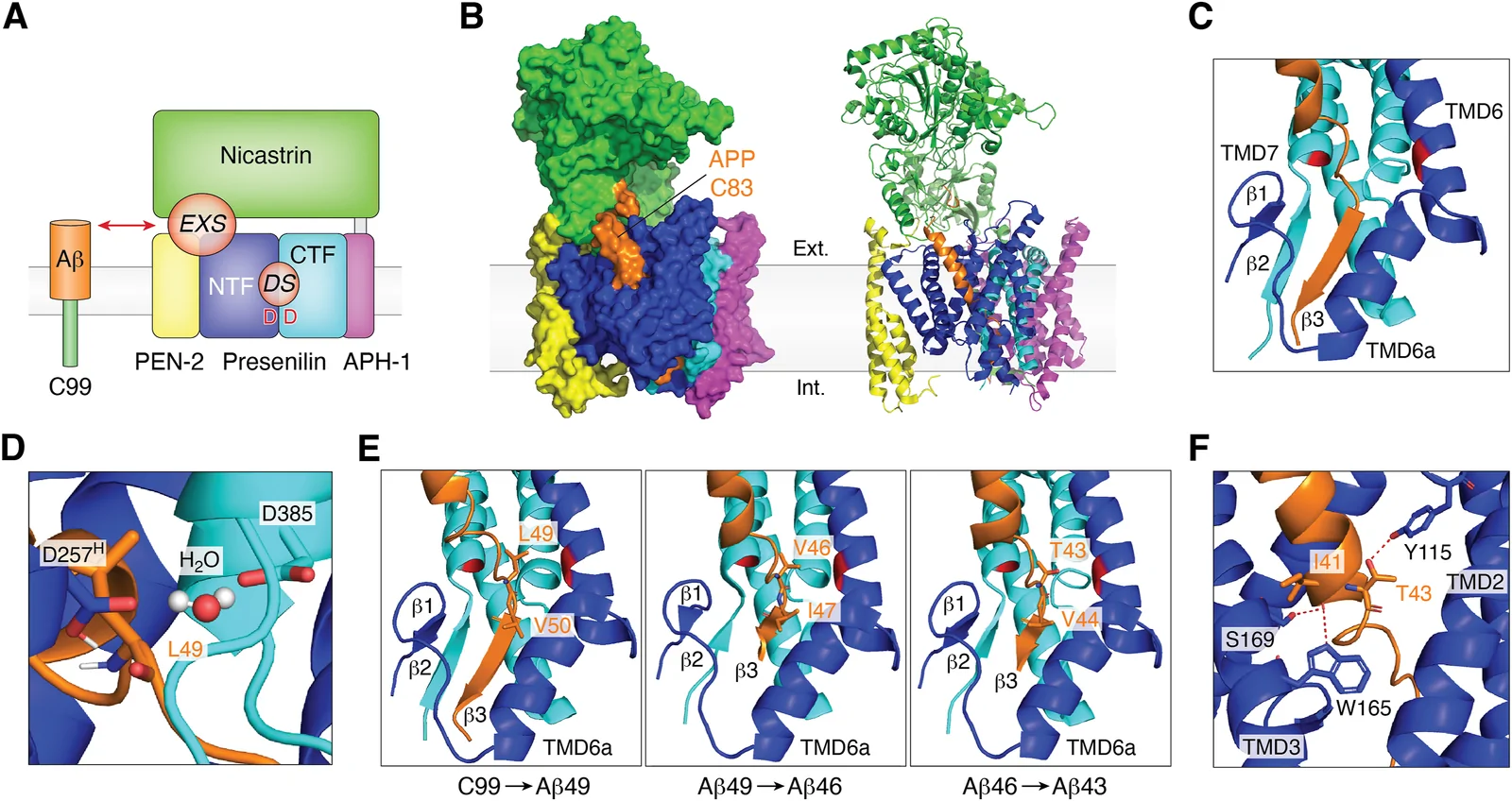
**Substrate recruitment and processing.***A*, substrate passage of C99 to the active site of γ-secretase. Exosite (EXS) and docking site (DS) regions are marked with red circles. *B*, Cryo-EM structure of γ-secretase in complex with APP C83 substrate (PDB 6IYC) in surface (*left panel*) and cartoon (*right panel*) representation. C83 is shown in *orange*. *C*, Magnified view of the hybrid β-sheet in the cryo-EM structure of γ-secretase in complex with APP C83 substrate. *D*, catalytically active geometry capable of APP C83 substrate cleavage at the L49 carbonyl by a water molecule in the D257-protonated state, based on (124). Active site aspartate residues of presenilin and L49 substrate residue are highlighted as sticks. *E*, magnified views of the hybrid β-sheet in the cryo-EM structures of γ-secretase in complex with APP C99 substrate (PDB 8X54), Aβ49 (PDB 8X52), and Aβ46 (PDB 8X53). Substrate residues comprising the respective scissile peptide bonds are highlighted in the structures as sticks. *F*, magnified view of C99 in the γ-secretase substrate-binding channel. Residues of presenilin forming H-bonds with the substrate are highlighted as sticks. TMDs of presenilin surrounding the substrate (*orange*) are indicated. In *C* and *E*, the β-strands of presenilin (β1, β2) and substrate (β3) forming the hybrid β-sheet and the interacting TMD6a of presenilin are indicated, as are the position of the catalytic residues (*red*) in TMDs 6 and 7.

Studies of substrate–enzyme interactions using chemical, structural, and molecular biology approaches have allowed mechanistic insights into Aβ generation, from substrate recruitment to cleavage. Photoaffinity labeling experiments using C99-based substrates, each of which contained a single photocrosslinkable amino acid at residue positions 1 to 68, provided insights into how substrates bind to the enzyme (110). This analysis showed that C99 interacts with exosites—substrate binding sites distant from the enzyme active site—located in NCT, PEN-2, and the presenilin NTF (Fig. 3*A*). These exosites are encountered by C99 before it interacts with the active-site region in the presenilin NTF/CTF heterodimer, suggesting that C99 moves to the active site in a stepwise manner. Impaired exosite binding by C99 leads to lower substrate levels at the active site, thereby reducing substrate cleavage (116). The substrate path from exosites to the active site may also be used by NOTCH1, which competes with C99 for binding to its exosites (110). The shorter APP CTF substrate C83, generated by α-secretase cleavage, also interacts with γ-secretase exosites but more weakly and differently from C99 (116). Interestingly, several peptides that inhibit Aβ generation and specifically bind to the N-terminal region of C99 (116, 117) interfere with its exosite interactions (116), suggesting that these binding sites could be targeted to selectively lower Aβ.

The existence of binding sites outside the active site had been proven earlier by pharmacological inhibition studies, which surprisingly showed that transition state analog inhibitors non-competitively inhibit γ-secretase (118). Furthermore, γ-secretase was found to remain in complex with a substrate even when its active site was occupied by a transition state analog inhibitor (119). The use of short substrate-mimicking peptide inhibitors as experimental tools allowed to identify one such exosite, the docking site, located at the presenilin NTF/CTF interface very close to the active site (120). Competition experiments showed that the docking site is distinct from the C99 exosites identified by photoaffinity labeling (110) (Fig. 3*A*).

### γ-Secretase generates Aβ through repeated conformational changes of the E–S complex

Following the high-resolution cryo-EM structures of γ-secretase (84, 85, 121), additional cryo-EM structures have been obtained with bound APP C83 (Fig. 3*B*) and NOTCH1 substrates (122, 123). Both substrate-bound holo-enzyme structures show substantial conformational changes compared to the unliganded apo-enzyme structure. Presenilin TMD2, which is unordered in the apo structure, becomes ordered, and TMD6 splits into TMD6a, an additional subhelix that extends TMD6 C-terminally. The substrates adopt an extended conformation around the ε-cleavage sites, followed by a β-strand (β3) that forms a hybrid β-sheet with two β-strands (β1 and β2) from presenilin (Fig. 3*C*).

In silico molecular dynamics (MD) simulations suggest that formation of the hybrid β-strand helps drain excess water from the active site (124). This drainage allows γ-secretase to adopt a catalytically active geometry for proteolysis. In this geometry, the active site aspartate residues, the substrate’s scissile bond carbonyl, and a water molecule are positioned in proper coordination for cleavage (Fig. 3*D*). Insights into the initial processing steps of C99 were also obtained from Gaussian-accelerated MD simulations, which revealed catalytically active geometries for both the initial ε-site cut and the subsequent first carboxy-terminal trimming step from Aβ49 to Aβ46 (125, 126).

Cryo-EM structural analyses of γ-secretase in complex with C99, Aβ49, Aβ46, and Aβ43 showed that Aβ is generated in a systematic fashion with the same molecular mechanism along the substrate processing path (127) (Fig. 3*E*). This work nicely showed that each sequential trimming step - from Aβ49 to Aβ46, Aβ46 to Aβ43, and Aβ43 to Aβ40 - is always accompanied by the formation of a hybrid β-sheet, causing the unfolding of three preceding residues of the APP TMD helix and allowing cleavage just upstream of the β-strand. As shown by these structures as well as by a cryo-EM structure of the PS1/APH-1b γ-secretase complex with Aβ46 in lipid-nanodiscs (128), the substrates are stabilized in the substrate-binding channel by the formation of several hydrogen bonds with presenilin (Fig. 3*F*). Consistent with their substrate-stabilizing role, removal of these bonds by mutating the corresponding presenilin residues leads to reduced processivity of γ-secretase and increased generation of longer Aβ42/43 (128).

In addition to the substrate-bound γ-secretase structures, atomic cryo-EM structures were also obtained for γ-secretase in the GSI-bound state (129). The structures showed that inhibitor binding induces conformational changes that lead to the formation of TMD6a and the β-strands β1 and β2 as in the substrate-bound states. Furthermore, the GSIs bind in the same region of the active site where the substrate adopts its β-strand conformation. GSIs mimicking the tetrahedral transition state of the substrate during cleavage interact with the two aspartate residues (129). Non-transition state-analog GSIs, including NOTCH1-sparing inhibitors, bind in the same region, but do not extend into the active site and form fewer stabilizing hydrogen-bond interactions (129). Differences in hydrogen-bond-mediated stabilization also appear to be the molecular basis of one of those inhibitors’ selectivity for PS1 over PS2 (86).

Overall, the formation of β-stranded conformations in substrate- and inhibitor-bound γ-secretase structures is in accordance with the fundamental principle that substrates and substrate-mimicking inhibitors bind in an extended conformation to the active site of proteases (130). This has also been observed for other intramembrane proteases (131, 132, 133).

## Determinants of γ-secretase processivity

### The APP N-terminal juxtamembrane domain influences γ-secretase processivity

**Figure 4:**
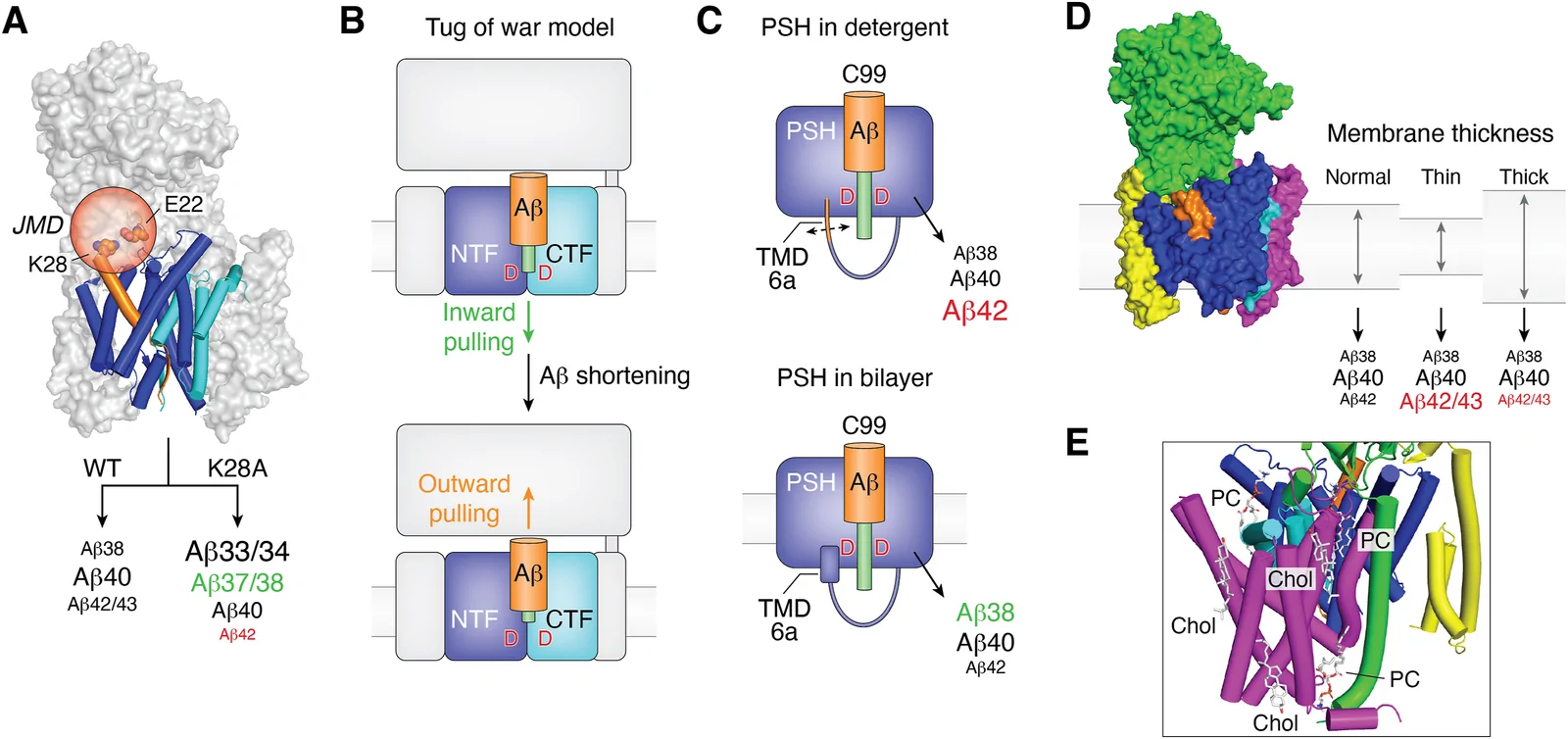
**Determinants of γ-secretase activity and Aβ length.***A*, processivity of γ-secretase (PDB 6IYC) and resulting length of Aβ species is influenced by hydrophilic residues in the JMD region (marked with a *red circle*) such as K28 or E22 (denoted as *spheres*. Increasing hydrophobicity by removal of the positively charged K28 in the K28A mutant has a particularly strong effect on the production of short Aβ species. *B*, in the “the tug of war” model, inward pulling forces facilitate substrate movement to the catalytic site. This continues during Aβ shortening until outward pulling forces become predominant, thereby enabling product release. *C*, processivity of PSH (*blue*) is enhanced in the lipid bilayer by the stabilization of TMD6a and the promotion of the formation of an active site geometry. In detergent (*top panel*), TMD6a is flexible and unstable and the catalytic aspartate residues are more distant to the substrate as compared to PSH in the bilayer (*bottom panel*). *D*, processivity of γ-secretase (PDB 8X54) and Aβ species produced are influenced by membrane thickness. *E*, cholesterol and PC lipids shown as sticks in the cryo-EM structure of γ-secretase in complex with C99 substrate (PDB 8X54). In *A*, *C*, and *D*, the font size used for the different Aβ species indicates their respective levels when generated from: (*A*) WT or K28A mutant APP substrates, (*C*) PSH in detergent or bilayer, or (*D*) membranes of different thicknesses. Beneficial Aβ species are indicated in *green* and pathological Aβ species in *red*.

After the initial cleavage at the ε-site, γ-secretase sequentially cleaves the APP TMD to release Aβ40 as the major cleavage product within a series of peptides ranging from Aβ37-43. Mutational analysis showed that the N-terminal juxtamembrane domain (JMD)—in particular the membrane-flanking positively charged K28 residue (numbering according to the Aβ sequence)—influences the length of the Aβ products produced (134, 135, 136, 137, 138). Hydrophobic substitutions at this position such as in the K28A mutant increase the processivity of γ-secretase, such that Aβ33 and Aβ34, which are not normally produced, become the end products of the Aβ product lines (Fig. 4*A*). When the APP substrate is bound to γ-secretase, K28 comes into close proximity to and interacts with the NCT ectodomain. Stabilizing this interaction by a salt bridge with the NCT I242E mutant increases γ-secretase processivity, suggesting that the NCT ectodomain contributes to determining the final length of the Aβ products (139). Systematic mutational analysis of the APP JMD further showed that increasing its hydrophobicity generally promotes the production of short Aβ species (≤Aβ34), whereas increasing its polarity limits the length of the Aβ products to longer forms (140). Interestingly, hydrophobic substitutions in the JMD can correct the γ-secretase processivity deficiencies caused by FAD mutations in the APP TMD and in presenilin and increase short Aβ generation even in this background. Based on thermoactivity analysis, hydrophobic K28 mutations stabilize the E–S complex, counteracting the destabilization imposed by FAD mutations. Very similar results were observed with mutations of the negatively charged D23 residue in the APP JMD. Charged polar residues in the JMD thus represent energy barriers that must be overcome to enable continuous threading of the substrate into the substrate-binding channel.

To explain how APP is processed in the substrate-binding channel as a function of the forces acting on the substrate, the “tug of war” model was proposed (140) (Fig. 4*B*). In this model, two opposing forces act on the substrate during its processing. One is an inward pulling force acting on the negatively charged substrate neo-C-terminus—presumably through interactions with nearby positively charged residues in presenilin, as suggested by MD simulations (141). The other is an outward pulling force acting on the JMD, caused by the unfavorable inward movement of K28 into the hydrophobic substrate-binding channel. Accordingly, the final length of Aβ depends on the relative contribution of these forces, which are influenced by mutations in the substrate and the enzyme. Under normal conditions, they largely balance each other out to form ∼80% Aβ40 as the major Aβ species. It should be noted, however, that the “tug of war” model is inconsistent with the observation that Aβ49 with a C-terminal amide is processed to shorter Aβ species (142).

### γ-Secretase activity is influenced by the membrane lipid environment

Given that γ-secretase is an integral membrane protein complex, it is evident that its activity should be directly influenced by the surrounding bulk lipids. Indeed, it has been shown that bulk membrane lipids are indispensable for γ-secretase to remain active. When γ-secretase is isolated from cells using a suitable detergent, such as the commonly employed CHAPSO, and purified, it remains stable as a protein complex but is inactive. Its activation requires reconstitution into phospholipid membranes (143). Unlike γ-secretase, its archaeal presenilin/SPP homolog (PSH) can cleave C99 in n-dodecyl β-D-maltoside detergent micelles. However, reconstitution of PSH into a phosphatidylcholine (PC) membrane bilayer stimulates the enzyme and increases its processivity (144) (Fig. 4*C*). Reconstitution of γ-secretase into liposomes composed of different glycerophospholipids further demonstrated that PC lipids are beneficial for activity, whereas phosphatidylethanolamine (PE), phosphatidylinositol and phosphatidylserine lipids as well as PE plasmalogens are inhibitory (145, 146, 147). The observed inhibition with PE was attributed by MD simulations to altered lipid-protein interactions with γ-secretase in this lipid environment (148).

When γ-secretase was reconstituted in PC membranes of varying thicknesses (*i.e.*, differing acyl chain lengths), an optimal activity for cleaving C99 was observed in membranes with a hydrophobic thickness of ∼29 Å (149), consistent with the predominant chain lengths of endogenous phospholipids associated with γ-secretase (150). Notably, an increase in membrane thickness was accompanied by an increased processivity of γ-secretase, leading to a reduced relative production of the toxic Aβ42 species (149) (Fig. 4*D*). This effect was corroborated in studies utilizing HEK293 cells enriched with very-long-chain fatty acids, such as erucic acid (151) and nervonic acid (152). It was demonstrated that these fatty acids were esterified to membrane lipids, remodeling the lipid membrane and resulting in an increased γ-secretase processivity. Short-chain fatty acids, on the other hand, have no direct effect on γ-secretase cleavage, although they affect Aβ deposition in mouse models (153).

Interestingly, phospholipid and cholesterol molecules have been identified in both substrate-free and substrate-bound cryo-EM structures of γ-secretase (85, 122, 123) (Fig. 4*E*). It has been suggested that these molecules stabilize the structure, but their interaction with γ-secretase may also indicate regulation of the enzyme by specific, tightly bound lipid species. This is supported by the finding that the presence of cholesterol increases the activity of purified γ-secretase reconstituted into lipid membranes (145). Furthermore, GM1 - the most common ganglioside in the brain - was also found to activate γ-secretase, probably through a direct interaction with PS1 (154). In addition, certain acidic cholesterol derivatives, such as the steroids 5β-cholanic acid and cholestenoic acid, have been reported to increase γ-secretase processivity, although their mechanism of action and molecular target remain unclear (155, 156).

## Strategies for targeting γ-secretase in AD

### GSIs have proven ineffective in AD clinical trials and cause significant side effects

The central role of γ-secretase in Aβ generation led to major efforts in identifying and developing potent GSIs for the treatment of AD. A number of such compounds were developed by pharmaceutical companies and entered AD clinical trials. Unfortunately, the inhibitors tested in the clinic failed, and one of them, semagacestat, even worsened cognitive decline (157). A common problem noted in the trials was their association with adverse effects such as gastrointestinal toxicity, loss of hair color, and skin cancer (157, 158). These adverse effects were at least partly due to inhibition of the Notch signaling pathway. In addition, acting by a substrate competition mechanism (140), semagacestat causes an accumulation of unprocessed long Aβ45/46 and the small peptide cleavage byproducts in the membrane, which could be toxic if not degraded (159). Since additional studies also showed membrane toxicity of APP CTFs in the endosomal/lysosomal pathway, it is also possible that accumulation of the uncleaved APP CTF substrates upon γ-secretase inhibition contributed to the side effects (160, 161). Although these setbacks led to the termination of GSI programs for AD, repurposing the inhibitors for cancers with dysregulated, excessive Notch signaling, such as in T-ALL, was considered a safe potential therapeutic strategy for these and other conditions with aberrant Notch signaling (13, 162). One such GSI, nirogacestat, demonstrated efficacy in a phase 3 clinical trial for the treatment of desmoid tumors and has been approved in the US as a systemic therapy for affected adult patients (163).

#### γ-Secretase modulators lower Aβ42/43 without affecting ε-site signaling cleavages

**Figure 5:**
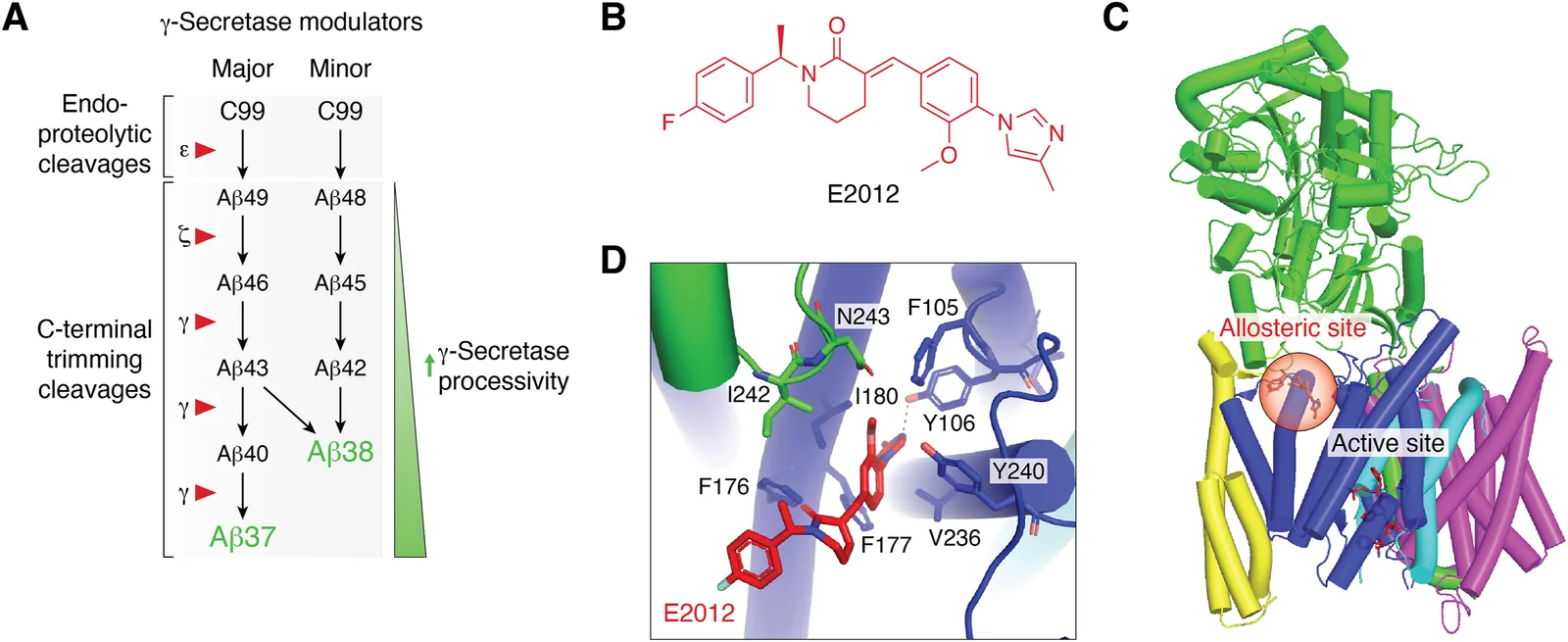
**Modulation of γ-secretase activity.***A*, GSMs cause an increased processivity of γ-secretase, leading to the accumulation of shorter Aβ forms (*green*), thereby reducing the longer pathogenic Aβ42 and Aβ43 species. *B*, structure of GSM E2012. *C*, cryo-EM structure of γ-secretase in complex with E2012 GSM bound at the allosteric site and L-685,458 GSI at the active site (PDB 7D8X). The region of the allosteric site is marked with a red circle; GSM and GSI compounds are shown as sticks. *D*, magnified view of E2012 (*red*) at its binding site; nearby interacting residues are shown as sticks.

Unlike GSIs, which block the overall activity of the enzyme and broadly interfere with substrate cleavage, another class of γ-secretase-targeting drugs, the γ-secretase modulator (GSM) compounds, act on the enzyme in a different manner. As first observed for a subset of non-steroidal anti-inflammatory drugs, GSMs shift γ-secretase cleavage from Aβ42 to Aβ38 without affecting the levels of substrate ICD formation resulting from the initial endoproteolytic ε-site cleavage (164, 165). Since the seminal observation that Aβ42 could be lowered without inhibiting overall γ-secretase activity, GSMs programs have been initiated by pharmaceutical companies to identify and develop such compounds into clinically relevant drug candidates (166). Advanced GSMs typically act on both Aβ product lines and promote the processivity of γ-secretase, converting the long species Aβ42 and Aβ43 to the shorter species Aβ37 and Aβ38 (111, 167) (Fig. 5*A*). Affinity-labeling experiments demonstrated that all these compounds target the NTF of presenilin at allosteric site(s) (168, 169, 170, 171, 172). Consistent with mutagenesis studies (173, 174, 175), a cryo-EM structure of one of these GSMs, E2012, in complex with γ-secretase in the presence of a transition state analog GSI revealed an extracellular binding site at the interface of presenilin hydrophilic loop 1 and NCT (129) (Fig. 5, *B*–*D*).

Unlike several earlier candidates that were discontinued for a variety of reasons, the most recent GSM, nivegacetor (also known as RG6289 or RO7269162), developed by Roche, has shown a favorable preclinical safety profile (176). The compound engaged its target in a phase 1 study, reducing Aβ42 and Aβ40 in plasma and CSF, while concomitantly increasing Aβ37 and Aβ38 (177). Based on these data and the clinical safety and tolerability profile of the drug, a phase 2 trial ('Gabriella'; NCT06402838) was started (178). The concept that GSMs should be safe and effective in AD is further supported by recent data showing that the shorter Aβ species inhibit the aggregation of Aβ42 (179, 180), and by a correlation between higher Aβ38 levels and slower disease progression in patients (181). Consistent with these observations, the CSF Aβ37/Aβ42 ratio was found to be the most useful marker in discriminating cognitively normal individuals from AD patients (108). Advanced GSMs should, in principle, also be effective in lowering Aβ42/43 generated by mutant γ-secretases in presenilin FAD mutation carriers (182, 183, 184). Indeed, in a phase 1b study involving a limited group of PS1 E280A mutation carriers, nivegacetor was found to be effective in lowering Aβ42 in plasma and CSF, while increasing Aβ37/38 (185).

Although GSMs are not expected to interfere with ICD-mediated signaling, they may modulate the composition of Aβ-like peptides of other substrates. This has been shown to be the case for NOTCH1 (186, 187) and it is well conceivable that this may also be the case for other γ-secretase substrates. If Aβ-like peptides may have biological functions that depend on their respective C-terminal peptide length, this could have therapeutic implications. With regard to Aβ itself, GSMs are not expected to interfere with its putative synaptic function in the brain, given that GSMs preserve total Aβ levels and do not impact the N-terminal Aβ region which is thought to underlie this function (54, 55).

## From γ-secretase substrate recognition to substratome

### γ-Secretase has diverse substrate requirements

Apart from the requirements of a short N-terminal ectodomain and the involvement of exosite-mediated initial binding to the enzyme, additional factors have been reported to play important roles in substrate recognition and cleavage. Besides a cleavable TMD, both the extracellular and intracellular JMDs must be permissive for cleavage (188, 189). In addition, the substrate TMD must not be palmitoylated (189). This post-translational modification could affect cleavage in several ways, including hindering the substrate to fit sterically into the substrate-binding site.

With regard to TMD cleavability, TMD backbone flexibility of the substrates—mediated in APP by residues G37/G38 acting as a hinge, located 10 amino acids upstream of the ε-cleavage sites—has been extensively studied with regard to its potential facilitation of TMD entry into the catalytic cleft (190, 191, 192). Remarkably, it was found that an APP-based substrate construct with a N-terminal poly-leucine stretch containing this flexible di-glycine hinge, combined with the native C-terminal TMD sequence of APP, was cleaved with similar efficiency as the native C99 substrate (193). In addition, it was shown that altered backbone dynamics upstream of the cleavage site might enhance helical unfolding of the cleavage region by cooperative interactions (192). Another study demonstrated that helix-stabilizing and -destabilizing mutations in the di-glycine hinge in the APP TMD had no impact on the helix stability around the ε-sites (191). Nevertheless, both mutations impaired the γ-secretase cleavage. The latter was explained by altered bending and twisting motions of the TMD, caused by the mutations of the di-glycine hinge. Both mutations result in different predominant conformations compared to WT, and both would affect the proper movement of the cleavage site region into the catalytic cleft in different ways (191).

Although helical unfolding around the cleavage site should enable the catalytic event and may be facilitated by local instabilities in the TMD helix of γ-secretase substrates (142, 194), the general picture that has emerged is that unfolding events around the ε-sites must be induced by a series of productive substrate-enzyme interactions, rather than by intrinsic backbone flexibility of the domain around the initial cleavage sites of the substrates. The requirement to form energetically favorable interactions with the enzyme during substrate recruitment allowing correct substrate positioning in the catalytic cleft may explain the markedly slow kinetics of γ-secretase substrate cleavage (195, 196). The relative contributions of the factors required for productive substrate-enzyme interactions remain, however, unclear and are not readily discernible from the sequences of different substrates.

### γ-Secretase substrates have a common physicochemical signature

Substrates of soluble proteases typically contain short sequence motifs around their cleavage sites that are recognized by these enzymes. For example, the initiator caspase of the intrinsic apoptosis pathway, caspase-9, cleaves its substrates after the aspartate residue in an ESDG cleavage site motif (197). In contrast to such soluble proteases, intramembrane proteases often lack identifiable cleavage site motifs (198, 199, 200, 201). While initial cleavage sites are known for more than 20 γ-secretase substrates, no clear recognition motifs can be deduced (202). Therefore, a long-standing question has been whether a common, yet unknown, property exists (203) as a unifying principle that allows γ-secretase to discriminate between substrates and other single-span transmembrane proteins that are not cleaved, referred to as non-substrates.

**Figure 6:**
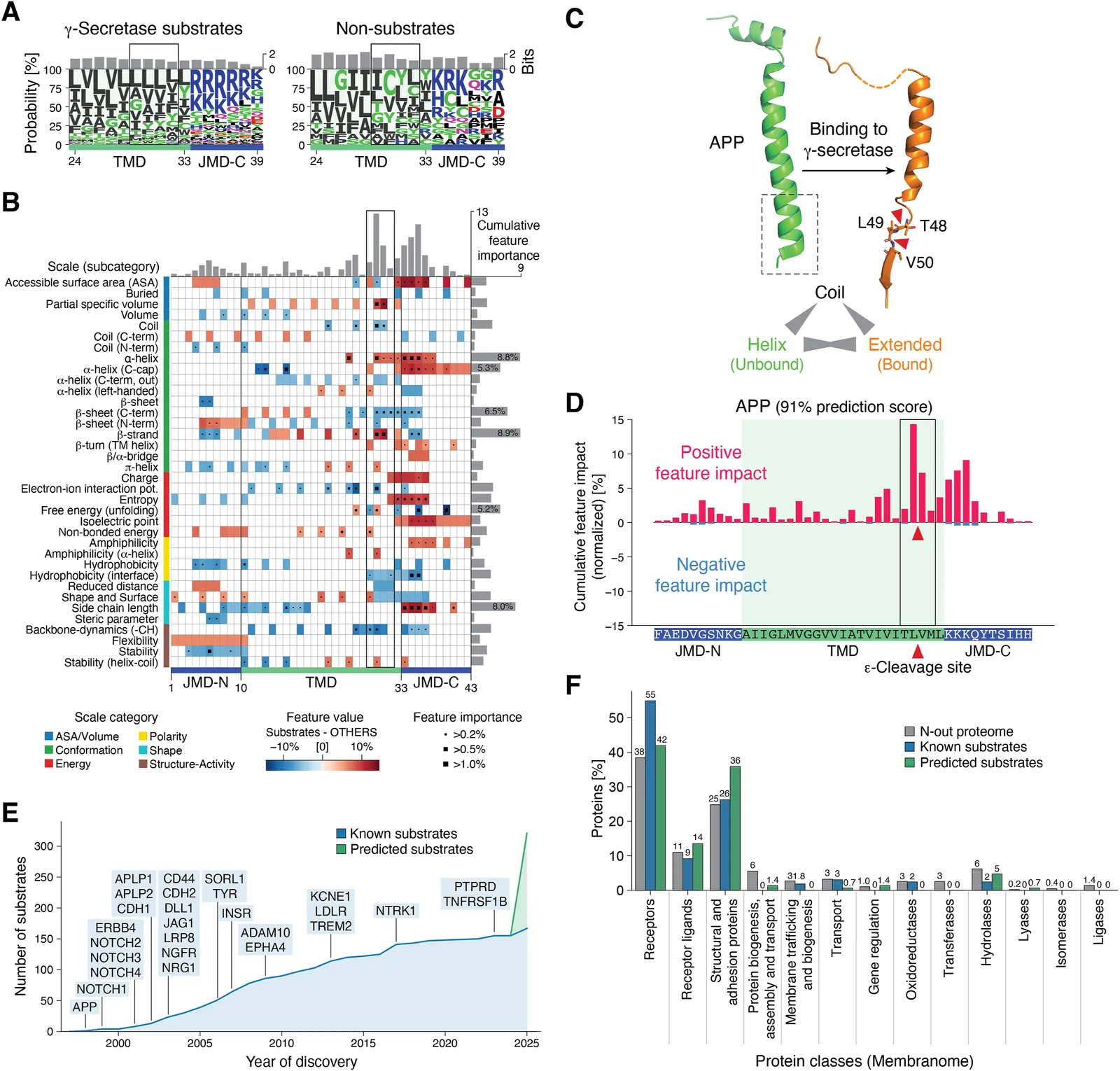
**Physicochemical signature of γ-secretase substrates and γ-secretase substratome.***A*, sequence logos for known γ-secretase substrates (*left*) and non-substrates (*right*), based on (202); TMD boundaries annotated with TMHMM (274). The bit score scale at the top indicates sequence conservation per position, measured as information content in bits, ranging from 0 (all 20 amino acids equally likely) to a theoretical maximum of ∼4.32 bits (log_2_ (20)); only one amino acid observed). *B*, CPP feature map showing the mean differences of feature values (substrates *versus* OTHERS) per residue position and AAontology scale subcategory (205), where OTHERS - used as the reference/background set - is a redundancy-reduced subset of the N-out proteome containing solely proteins with unknown substrate status (202). Gray bars indicate cumulative feature importance per subcategory (*right*) and residue position (*top*). *Black squares* mark positions with feature importance above 0.2%. Total TMD-JMD sequence length was set to 43 amino acids to match the TMD-JMD sequence length of APP. *C*, structural snapshots of APP in the unbound helical state (PDB 2LLM, *green*) and in the γ-secretase-bound extended state in which the ε-cleavage sites (*red arrows*) are exposed and a β-strand spanning the TMD/intracellular interface region is formed (PDB 6IYC, *orange*). The C-terminal part of unbound APP undergoing conformational transition upon binding is indicated with a dashed box. Schematic (*bottom*) of the conformational equilibrium between coil, helix, and strand, highlighting the helix-to-strand transition from unbound (*green*) to bound (*orange*) states. Residues of the ε-cleavages sites are shown as sticks. *D*, CPP-SHAP profile for APP, showing cumulative feature impact (positive in *red*, negative in *blue*) per residue position across its JMD and TMD regions. The ε-cleavage site (for the major product line starting with Aβ49) is marked by an arrow. In *A*, *B*, and *D*, each *black box* highlights the same sequence region around the ε-cleavage site. Sequence logos (*A*), CPP feature map (*B*), and CPP-SHAP profile (*D*) were created using AAanalysis (275) v1.0.3. *E*, Timeline of γ-secretase substrate discovery including known (experimentally validated) and computationally predicted substrates (202). The cumulative number of substrates is shown for known (*blue*) and predicted (*green*) substrates. Key substrates (selected for historical, mechanistic, or clinical significance) are highlighted with the year of discovery. *F*, distribution of functional protein classes as defined by their molecular function in the Membranome database (209) (retrieved January 20, 2025) shown for all Membranome-annotated proteins across three protein groups: the human N-out proteome (*gray*), known substrates (*blue*), and predicted substrates (*green*). Bars represent the relative proportion of protein classes within each protein group, with numbers above bars indicating percentages rounded to the nearest one or to the nearest 10th for values below 2.

Patterns for discriminating substrates from non-substrates (Fig. 6*A*) can be identified computationally using machine learning models (204). In addition to experimentally validated data, these models require properties, called “features,” to train the machine learning algorithms. A recent study by Breimann *et al.* (202) introduced the sequence-based bioinformatics algorithm Comparative Physicochemical Profiling (CPP), which reveals the most discriminative features between two sets of protein sequences, and applied it to identify γ-secretase substrates. By comparing γ-secretase substrates with other single-span type I membrane proteins, CPP identified over 100 discriminative features.

Using amino acid property scales curated in AAontology (205), these features capture physicochemical properties (*e.g.*, volume, charge, and hydrophobicity) within distinct amino acid segments or patterns within the substrates´ TMD and their flanking JMD regions. Providing a new conceptual framework, a physicochemical signature (Fig. 6*B*) of γ-secretase substrates was identified which unveiled three key feature groups. First, the region surrounding the initial cleavage site favors α-helical and β-strand over coil conformations. This aligns with the experimentally observed helix-to-strand transition between unbound and bound γ-secretase substrates, as shown for APP (Fig. 6*C*) (122) and NOTCH1 (123). Second, residues with long side chains, high surface area, and increased disorder are abundant in the first four positions of the C-terminal JMD. These residues act as a membrane anchor at the intracellular membrane-water interface, which is a characteristic common to single-span transmembrane proteins in general (206) but more pronounced in γ-secretase substrates. Third, small residues within the N-terminal TMD region, crucial for backbone flexibility—especially for APP owing to its GG hinge (191)—are more prominent in γ-secretase substrates. Overall, this study demonstrated that substrate-defining properties of γ-secretase can be described by a physicochemical signature present within a specific sequence area of around 40 residues, including the initial cleavage site. Moreover, based on these findings substrate prediction scores could be calculated for individual substrate/non-substrate proteins.

The agreement of the identified features with experimental findings suggests that they capture common traits among γ-secretase substrates. However, the contribution of specific features varies across substrates. By combining CPP with a general explainable AI method, SHAP (SHapley Additive exPlanations) (207), the impact of individual features on substrate prediction scores was quantified at single-residue resolution. SHAP assigns each feature a contribution value that reflects its positive or negative influence on a given prediction, thereby making the model's decision interpretable. For APP (Fig. 6*D*), the high prediction score (91%) (202) was driven predominantly by positive contributions of features around the initial cleavage site and the C-terminal anchoring region. As was also shown for other proteins, such CPP-SHAP analyses provide an important integrative framework for understanding why a protein is a γ-secretase substrate or not (202). It should be noted, however, that these prediction scores reflect the physicochemical signature captured by the model and require experimental validation to confirm actual cleavage.

### The γ-secretase substratome: Known and predicted substrates of γ-secretase

Following the identification of presenilins as the enzymes responsible for the catalytic activity of the APP-cleaving γ-secretase in 1998 to 2000 (63, 64, 67, 68, 70, 71, 72, 73, 74, 75, 76, 77, 78), substrate discovery has progressed steadily (Fig. 6*E*). Key substrates followed soon after: the cancer-associated Notch receptors (NOTCH1–4); the receptor tyrosine kinase ERBB4 of the EGFR family; the adhesion proteins cadherin 1 (CDH1) and cadherin 2 (CDH2); Notch receptor ligands such as Jagged 1 (JAG1); the EGFR family ligand neuregulin 1 (NRG1); the adhesion receptor CD44; the nerve growth factor receptor (NGFR); and, more recently, the AD-associated lipoprotein receptor LDLR and the microglial receptor TREM2. Over nearly 3 decades, ∼150 substrates were validated across diverse protein classes (12). Yet, this represents merely 10% of the ∼1500 human single-span transmembrane proteins with an extracellular N-terminus—the so-called “N-out proteome”—that constitute the potential substratome of γ-secretase.

To systematically explore this space, Breimann *et al.* (202) leveraged the physicochemical substrate signature to train machine learning classification models. Each protein of the N-out proteome received a substrate prediction score, ranging from 0% (non-substrate) to 100% (substrate). A total of 250 high-confidence (HC) substrates (≥80% prediction score, 16.3% of the N-out proteome) were predicted, including 160 novel candidates. Five of these (ERBB2, CD2, CD68, CD86, and FAM174A) and six additional low-confidence substrates (50–80% prediction score; GPNMB, PCDH17, ICAM1, CLMP, STYK1, and TIMD4) were experimentally validated. Together with a recent large-scale proteomics screen in microglia-like cells (208), the number of experimentally validated γ-secretase substrates now approaches 170. Combined with the 150 unvalidated HC predictions, the putative substratome expands to ∼320 proteins (Fig. 6*E*). Importantly, not all predicted candidates are expected to be *bona fide* substrates, as cleavage also depends on determinants such as subcellular localization and ectodomain shedding, as well as cell-type specific co-expression of substrate and protease.

For a systematic overview, all known and predicted substrates, together with all proteins of the N-out proteome, can be classified into 13 functional protein classes using the Membranome database (209) (Fig. 6*F* and Table 1). Among known substrates, the dominant protein classes were receptors, structural and adhesion proteins, and receptor ligands with very small contributions from transport proteins and membrane trafficking proteins. Enzyme-related substrates were rare, limited to oxidoreductases and hydrolases. Main protein families included immunoglobulin (Ig)-like receptors (*e.g.*, CD86), receptor tyrosine kinase (RTK) proteins (*e.g.*, RYK), cadherins (*e.g.*, CDH1), and Ig superfamily proteins (*e.g.*, VCAM1). Transport protein groups were subunits of ion channels for calcium (CACHD1), potassium (KCNE1/2), and sodium (SCN1B/2B). An interesting class is that of receptor–ligand families in which γ-secretase processes receptors and their cognate ligands, as observed for the EGFR–NRG, EPH–ephrin, NOTCH–DLL/JAG, and plexin–semaphorin pairs (Table 1). Interestingly, comparing the known γ-secretase substrates with the entire N-out proteome, the former contained relatively more receptors (Fig. 6*F*).

**Table 1:** Protein classes of known and predicted γ-secretase substrates

Protein classes	Protein families	Substrates (known)		Substrates (predicted)	
Receptor | *Ligand* Pairs	EGF family	ERBB2, ERBB4	*BTC, Nrg1, Nrg2*	EGFR	*AREG*
	Ephrin family	EPHA2, Epha4, EPHA5, EPHA7, Ephb2, EPHB3, EPHB4, EPHB6	*Efnb1, Efnb2*	EPHA1, EPHB1	
	Notch family	Notch1, Notch2, Notch3, Notch4	*DNER, Dll1, JAG2, Jag1*		*DLL3*
	Semaphorins	PLXDC2		PLXNA1, PLXNA2, PLXNA4, PLXNB1, PLXND1	*SEMA4C, SEMA4F*
Receptors	APP family	Aplp1, APLP2, APP			
	Ig-like receptors	AGER, BSG, CD2, CD300A, CD86, Dcc, Ghr, IFNAR2, IL11RA, IL1R1, IL1R2, IL6R, MILR1, NEO1, PRTG, ROBO1, SIRPA, TIMD4, TREM2		BTNL9, CD274, CD276, ERMAP, FCAMR, FCGR2A, FCGR2C, HAVCR1, LILRB1, LILRB2, LILRB3, LILRB5, PIGR, PILRA, PILRB, ROBO2, ROBO3, SLAMF1, SLAMF6, TIGIT, TREML1, UNC5A, UNC5B, UNC5C, VSIR	
	LDL receptors	LDLR, LRP1, Lrp2, LRP1B, LRP6, Lrp8, Vldlr		LRP4, MAMDC4	
	LRRC family	Lrrc4b, VASN		LRRC24, LRRC4, LRRC4C, SLITRK1	
	Other receptors	CD44, DAG1, PKHD1		FAS, IL17RD, RAMP3	
	PTP superfamily	PTPRD, PTPRF, Ptprk, Ptprm, PTPRT, Ptprz1		PTPRA, PTPRE, PTPRH, PTPRJ, PTPRO	
	RTK superfamily	AXL, Csf1r, ERN1, ERN2, FGFR4, FLT1, FLT4, Fgfr3, IGF1R, INSR, MERTK, MET, MUSK, NTRK1, Npr3, Ntrk2, PTK7, Ryk, Styk1, TIE1, TYRO3		ACVRL1, DDR1, GUCY2C	
	Scavenger receptors	Cd68, CXCL16		CD163, LAMP1, LAMP3, MEGF10, MEGF11, MRC1, MRC2	
	Syndecan receptors	SDC1, SDC2, SDC3		SDC4	
	TNF receptors	NGFR, Nradd, TNFRSF17, TNFRSF1A, TNFRSF1B, TNFRSF21, TNR12		CD27, TNFRSF13C, TNFRSF19	
	VPS10 domain-containing receptors	SORCS1, SORL1, SORT1		SORCS2	
Receptor ligands	HLA antigens	HLA-A		CD1B, FCGRT, HFE, HLA-DPB1, HLA-E, HLA-F, HLA-H	
	Ig superfamily	CD200		CD79A	
	Neuroligins	Nlgn1, Nlgn2		NLGN3, NLGN4X, NLGN4Y	
	Other ligands	CX3CL1, TMEFF2		CRIM1, ELFN1, ELFN2, KITLG, SUSD2	
Structural and adhesion proteins	Cadherins	Cdh1, CDH2, CDH5, CDH6, CLSTN1, CLSTN2, CLSTN3, DSG2		CDH3, CDH4, CDH7, CDH9, CDH10, CDH11, CDH12, CDH15, CDH17, CDH18, CDH20, CDH22, CDH24, CDHR2, FAT3	
	Ig superfamily	CLMP, CXADR, Cadm1, Cspg4, Dscam, Dscaml1, Icam1, L1cam, Nectin1, Nectin2, Nectin3, SCN2B, Scn4b, VCAM1		CD22, CD226, CHL1, KIRREL1, MCAM, MXRA8, NFASC, SIGLEC16, SIGLEC5, SIGLEC6, TMIGD1	
	Neurexins	Nrxn1, Nrxn2, NRXN3			
	Other adhesion proteins	CD99, EPCAM, GLG1, LRRN3, MUC1, PDPN, SPN, Tacstd2, Tgfbr3		CRB3, DCBLD1, LRRN1, MUC17, ZP3	
	Other ligands			SELL, SELP	
	Protocadherins	PCDH12, PCDH17, Pcdha4, Pcdhga1, Pcdhga3, Pcdhgc3		PCDH1, PCDH11X, PCDH11Y, PCDH9, PCDHGA2, PCDHGA4, PCDHGA5, PCDHGA6, PCDHGA7, PCDHGA8, PCDHGA9, PCDHGA10, PCDHGA11, PCDHGA12, PCDHGB1, PCDHGB4, PCDHGB5, PCDHGB6	
	SEZ6/CSMD family	Sez6, SEZ6L, SEZ6L2		CSMD1, CSMD2	
Protein biogenesis, assembly and transport	Other biogenesis proteins			IGF2R, ITPRIPL2	
Membrane trafficking and biogenesis	Other membrane trafficking proteins	GPNMB, PMEL, SYT7			
Transport	Channel subunits	Cachd1, KCNE1, KCNE2, Scn1b, Scn3b			
	Other transport proteins			NPTN	
Gene regulation	Other gene regulation proteins			JTB, PARM1	
Oxidoreductases	Other oxidoreductases	Pam			
	Tyrosinases	Dct, Tyr, Tyrp1			
Hydrolases	Metalloproteases	ADAM10		ADAM7, ADAM8, ADAM12, MMP16	
	Neuropathy target esterases			PNPLA6, PNPLA7	
	Other hydrolases	ACP2, KL, Prima1		PRRG2	
Lyases	Other lyases			CA14	
Undefined function	Undefined function	FAM174A		C3orf18, SMIM29	

Known substrates were sourced from the most recent review (12) and a comprehensive prediction study (202). Predicted substrates were taken from the latter and limited to the reported HC substrate class set (prediction score ≥ 80%). Protein classes and families were adapted from the Membranome database (209) (retrieved January 20, 2025) with minor adjustments; additionally, we introduce a “Receptor | *Ligand* Pairs” class to highlight cases where γ-secretase processes both the transmembrane receptors and their cognate ligands. Gene names are from UniProt and follow the convention of UPPERCASE for human and Capitalized for non-human entries, following (12).

Comparing known substrates with predicted substrates, the latter showed a shift toward higher fractions of structural and adhesion proteins (dominated by cadherins and protocadherins), receptor ligands, and hydrolases. Notably, three functional classes absent from known substrates were added: protein biogenesis, assembly and transport (IGF2R, ITPRIPL2), gene regulation (JTB, PARM1), and lyases (CA14). In addition, within the class of hydrolases, four metalloproteases (ADAM7, ADAM8, ADAM12, and MMP16) expanded this enzyme-related protein family, which previously contained only ADAM10. Overall, the γ-secretase substratome is dominated by receptors, receptor ligands, as well as structural and adhesion proteins.

## The functional scope of γ-secretase

Unveiling the complete set of substrates of a protease—its substratome—is crucial for understanding its functional scope in health and disease. In their functional bioinformatics analysis, Breimann *et al.* (202) revealed a strong enrichment among the 250 HC substrates, compared to the N-out proteome, in functions such as cell communication, semaphorin interaction, caspase activation, or cancer- and immune-related pathways. New disease associations for γ-secretase were also identified, including terms related to type I diabetes mellitus and autoimmune thyroid disease. Network analysis further showed that HC substrates act as hubs, bottlenecks, or module members, underscoring their central role in biological systems.

The functional scope of γ-secretase is defined by what it cleaves and what this cleavage does - initiating, terminating, or modulating a specific function. Two functional levels can be distinguished: cellular processes (*e.g.*, extracellular function or signaling) and biological systems (*e.g.*, organs, cell types, or diseases). Associations between known γ-secretase substrates and functional terms are shown for cellular processes in Table 2 and for biological systems in Table 3. Here, each term–substrate association indicates that γ-secretase cleavage is functionally linked to a specific cellular or biological context; for example, substrate cleavage can trigger ICD-dependent transcription as in Notch signaling or dysregulated cleavage can contribute to disease as in aberrant APP processing in AD. While some term–substrate associations represent causal links supported by well-defined underlying cellular or biochemical mechanisms, others are best viewed as correlative associations: cleavage and a functional association are documented, but the complete underlying mechanism of how the associated cellular or biological functions are influenced by γ-secretase cleavage remains unclear.

**Table 2:** Functional scope of γ-secretase in cellular processes

Cellular function	Cellular process	Substrates
Extracellular Function	Cell adhesion	APP (228), ADAM10 (276), CADM1 (277), CDH1 (214), CDH2 (216), DSCAM (268), EPCAM (278), HLA-A (279), L1CAM (280), PCDH12 (238), PTPRK (281), PTPRM (281), SCN1B (269), SCN2B (282)
	Cell interaction	APP (228), CDH1 (214), DLL1 (283), Notch (210)
	Cell migration	APP (228), MET (284), SCN1B (269), SCN2B (282)
	Exocytosis	SYT7 (285)
	Phagocytosis	CD44 (286), LRP1 (286), TREM2 (287)
Intracellular Function	Cell proliferation	PTK7 (288)
	Cholesterol uptake	LDLR (211), LRP1 (211), LRP8 (211)
	Cytoskeletal dynamics	CDH1 (214), EFNB1 (224), PDPN (289)
	Endocytosis	LDLR (211), LRP1 (211), LRP2 (290), LRP8 (211), FGFR3 (266)
	Metabolism (Glucose)	CD44 (218)
	Metabolism (Lipid)	CD44 (218), TREM2 (225)
	Protein sorting	SORCS1 (242), SORL1 (242), SORT1 (242)
	Regulation of calcium channel	CACHD1 (291)
	Regulation of potassium channel	KCNE1 (245), KCNE2 (245)
	Regulation of sodium channel	SCN1B (269), SCN2B (269), SCN4B (269)
	Transcription	APP (228), CD44 (218), ERBB4 (221), NEO1 (292), Notch (210)
Signaling	Signaling (RTK)	AXL (223), CSF1R (221), RYK (221), TYRO3 (223)
	Signaling (RTK, ERBB)	ERBB4 (221), NRG1 (293)
	Signaling (RTK, Insulin)	INSR (294)
	Signaling (Chemokines)	CXCL16 (246), CX3CL1 (246)
	Signaling (FGF)	SDC3 (295)
	Signaling (JAK–STAT)	GHR (296), IFNAR2 (215)
	Signaling (MAPK)	CD44 (218)
	Signaling (NF-κB)	NGFR (297), TNFRSF1A (219)
	Signaling (Notch)	BSG (298), DLL1 (210), DNER (299), JAG1 (210), Notch (210)
	Signaling (Rho GTPase)	EPHA4 (212), PTPRD (241)
	Signaling (TGF-β)	TGFBR3 (300)
	Signaling (Wnt)	APP (301), CDH1 (214), LRP6 (302), PCDHGA1 (238), PCDHGA3 (238), RYK (303)
Cell growth	Cell cycle	CD44 (304), NOTCH1 (210)
	Cell growth	AXL (223), BTC (305), ERBB4 (221), JAG1 (210), MET (221), MUSK (223), TYRO3 (223)
	Cell proliferation	AXL (223), CD200 (306), HLA-A (279), MUSK (223), TYRO3 (223)
Cell differentiation	Differentiation	APP (228), CD44 (251), DLL1 (210), ERBB4 (221), JAG1 (210), Notch (210), RYK (221)
Cellular aging	Anti-aging	KL (307)
	Senescence	CD44 (304)
Cell death and cell survival	Apoptosis	APP (228), CDH1 (214), HLA-A (279), NGFR (308), NOTCH1–4 (210), TNFRSF1A (219)
	Autophagy	NOTCH1 (309)

Cellular functions of known substrates from Table 1 were grouped into seven categories: extracellular function, intracellular function, signaling and four categories spanning the cell lifespan: cell growth, cell differentiation, cell aging, and cell death. Cellular process terms were selected within each cellular function category to align with the Gene Ontology (GO) (310) nomenclature, and known substrates associated with the respective terms were identified by literature review. The links of the terms to γ-secretase can be either causal (*i.e.*, functional association with underlying mechanism, such as ICD release or CTF turnover) or correlative (*i.e.*, documented association without a clearly defined underlying functional mechanism). Each process term lists a representative, non-exhaustive set of substrates. For simplicity, substrates are given by gene name in upper case letters and may appear in multiple categories due to pleiotropy. “Notch” denotes cases where the cited publication does not clearly distinguish among the four Notch paralogs for the respective substrate–term association.

**Table 3:** Functional scope of γ-secretase across biological systems

Biological system	Term–substrate association
Cardiovascular system	*Angiogenesis* APP (231), CDH5 (235), EFNB2 (235), EPHB4 (235), ERBB4 (231), FLT1 (231), IGF1R (231), NOTCH1 (231); *Arteriogenesis* DLL1 (311); *Heart development* NOTCH1 (210); *Vascular development* NOTCH3 (210), NOTCH4 (210), TIE1 (312), *Cardiac excitability* SCN1B (269)
	Cardiomyocytes KCNE1 (245); Endothelial cells APP (229), CDH5 (236), Notch (210)
	Atherosclerosis LDLR (272); Pulmonary arterial hypertension NOTCH1 (210), NOTCH3 (210)
Digestive system	*Cholesterol metabolism* LDLR (272), LRP1 (211); *Fetal liver* JAG1 (312); *Liver* APP (313), JAG1 (210), NOTCH2 (210); *Stomach* JAG2 (314), NOTCH1 (314)
	Hepatocytes LDLR (272)
	Cancer (Colorectal) CD44 (218), DCC (315), NOTCH1 (210); Cancer (Gastric) LRP8 (258); Pancreatitis JAG1 (210), NOTCH1 (210)
Endocrine system	Beta cells BTC (305)
	Cancer (Thyroid) CD44 (256); Diabetes mellitus AGER (316), APP (273), CD44 (218), INSR (317)
Exocrine system	*Mammary gland* CDH1 (233), ERBB4 (221)
	Cancer (Salivary gland) NOTCH1 (210), NOTCH2 (210)
Immune system	*Bone marrow* Notch (318), TNFRSF17 (319); *Immune response* AGER (320); Notch (210), SIRPA (321), TREM2 (319); *Interferon response* CD44 (252); *Inflammatory response* AGER (320), CSF1R (320), CX3CL1 (246), CXCL16 (246), IL1R1 (247), IL1R2 (248), LRP1 (322), SPN (323), TNFRSF1A (320); *T-cell development* DLL1 (324), HLA-A (279), JAG1 (324), NOTCH1 (226); *Thymus* NOTCH1 (318)
	Dendritic cells DLL1 (210), NOTCH2 (210); Fibroblasts CD44 (252); Lymphocytes Notch (318); Macrophages CD44 (252), CSF1R (221), MERTK (325), SIRPA (321), TREM2 (320)
	Autoimmune disease TNFRSF17 (34); T-ALL NOTCH1 (210), NOTCH3 (210); Tumor-associated macrophages (TAM) Notch (210)
Integumentary system	*Hair* NOTCH1 (326), NOTCH2 (326); *Pigmentation* DCT (250), PMEL (327), TYR (250), TYRP1 (250); *Skin* NOTCH1 (326), NOTCH2 (326); *Wound healing* SDC1 (249)
	Epithelial cells CDH1 (214), CXADR (328), ERBB4 (221), MET (329), Notch (210); Melanocytes DCT (250), NOTCH1 (330), NOTCH2 (330), PMEL (327), TYR (250), TYRP1 (250)
	Cancer (Melanoma) AXL (254), LRP8 (258); Cancer (Skin) Notch (210); Desmoid Tumor Notch (210)
Muscular system	*Smooth muscle* NOTCH1–4 (331)
	Myocytes Notch (210)
Nervous system	*Axon guidance* APP (228), EPHB2 (332), PTPRD (241), PTPRF (241), ROBO1 (333); *Brain development* APP (228), LRP6 (302), SCN1B (269); *Central nervous system* APLP1 (228), APLP2 (228), APP (228), NOTCH1–4 (227), PTPRZ1 (334); *Neurogenesis* APP (228), ERBB4 (271), NOTCH1 (227), NOTCH2 (227), NRG1 (271), NRG2 (335), RYK (303); *Neuronal development* L1CAM (280), NOTCH1 (227), NOTCH2 (227), PCDH17 (238), PCDHA4 (237) *Neuronal differentiation* APP (228), LRRN3 (259), RYK (221); *Neuronal excitability* SCN2B (244); *Neuronal plasticity* APP (228), HLA-A (279); *Neuronal survival* APP (228), NGFR (243) *Neuronal trafficking* SORCS1 (242), SORL1 (242), SORT1 (242); *Synapse formation* APLP2 (228), APP (228), EPHA4 (212) NECTIN1 (239), NECTIN3 (239), NLGN1 (336), NRG1 (293), NRXN1 (240), NRXN2 (240), PTPRT (337); *Synapse function* APLP2 (228), APP (228), CDH2 (234), LRRC4B (338), NLGN1 (336), NRXN1 (240), NRXN2 (240); *Synaptic development* LRRC4B (338), PCDH17 (238), PCDHA4 (237); *Synaptic transmission* APLP2 (228), APP (228), CDH2 (234), DCC (217)
	Astrocytes NOTCH1 (227) NOTCH2 (227) Microglia CSF1R (320), TNFRSF1B (208), TREM2 (320); Neurons APLP1 (228), APLP2 (228), APP (228), CLSTN1 (339), CLSTN2 (339), CLSTN3 (339), L1CAM (280), NOTCH1 (227), NOTCH2 (227), SEZ6 (340); Oligodendrocytes CSPG4 (341), ERBB4 (221), LRP2 (342); Schwann Cells ERBB2 (343) NRG1 (343)
	Age-related macular degeneration APP (344); Amyotrophic lateral sclerosis NOTCH1 (227) NOTCH2 (227); Alzheimer's disease ADAM10 (276), APP (7), CLSTN3 (345) LDLR (211), LRP1 (211), LRP8 (211), SORL1 (242), TREM2 (270); Cancer (Neuroblastoma), LRRN3 (259); Cancer (Glioma) CSPG4 (346), ERBB4 (260), NOTCH1 (210), NOTCH2 (210); Down syndrome DSCAM (268); Epilepsy SCN1B (269); Frontotemporal dementia TREM2 (270); Nasu-Hakola disease TREM2 (270); Multiple sclerosis JAG1 (210), SCN2B (269); Parkinson's disease APP (347), TREM2 (270) Schizophrenia ERBB4 (293), NOTCH4 (210), NRG1 (271)
Reproductive system	Cancer (Breast) CDH1 (261), ERBB2 (262), ERBB4 (221), JAG1 (210), LRP8 (258), NOTCH1 (210), NOTCH4 (210); Cancer (Ovarian) NOTCH1 (210), NOTCH3 (210); Cancer (Prostate) CD44 (257), DAG1 (263), JAG1 (210), LRP8 (258), Notch (210), TACSTD2 (264)
Respiratory system	*Lung* ERBB4 (221), Notch (226)
	Goblet cells Notch (226)
	Cancer (Lung) AXL (255), LRP8 (258), NOTCH1 (210), NOTCH3 (210)
Skeletal system	*Bone growth* FGFR3 (266); *Cartilage* CD44 (253) TIE1 (312)
	Chondrocytes CD44 (253); Osteoblasts APP (348)
	Osteoarthritis CD44 (349), JAG1 (210), Nasu-Hakola disease TREM2 (270)
Urinary system	*Filtration* NOTCH1 (210); *Kidney* NOTCH1 (350), PKHD1 (351); *Reabsorption* LRP2 (232)
	Autosomal recessive polycystic kidney disease PKHD1 (351); Cancer (Bladder) CDH2 (265), FGFR3 (266)

Biological systems follow a standard organ-system taxonomy adapted from the MeSH Anatomy tree (https://meshb.nlm.nih.gov/treeview) and were edited for brevity. For term–substrate associations within each biological system, biological terms were grouped into biological processes and organs (italic), cell types (underlined), and diseases (normal typeface). Each term lists a representative, non-exhaustive set of substrates. Substrates are given by gene name in uppercase letters. “Notch” denotes cases where the cited publication does not clearly distinguish among the four Notch proteins for the respective substrate–term association. For clarity, the following list provides gene names in alphabetical order with alternative names in parentheses, included where only the latter were reported in the cited references within Tables 2 and 3: AGER (RAGE), BSG (CD147), CDH1 (E-cadherin), CDH2 (N-cadherin), CDH5 (VE-cadherin), CLSTN1 (Alcα), CLSTN2 (Alcγ), CLSTN3 (Alcβ, CST-3), CSPG4 (NG2), CXADR (CAR), DAG1 (dystroglycan), ERBB2 (HER2), FLT1 (VEFGR-1), HLA-A (HLA-A2), INSR (IR), L1CAM (L1), LRP2 (megalin), LRP8 (ApoER2), LRRC4B (NGL-3), LRRN3 (NLRR3), NEO1 (neogenin), NGFR (p75 neurotrophin receptor), SORL1 (SorLA), SORT1 (sortilin), SPN (CD43), TACSTD2 (Trop2), TNFRSF1A (TNFR1), TNFRSF17 (BCMA).

### The functional scope of γ-secretase in cellular processes

The known γ-secretase substrates are associated with numerous cellular functions and play critical roles in signaling and in processes across the cell life span - including cell growth, differentiation, aging, and death (Table 2). Very well documented is the involvement of γ-secretase in intracellular signaling pathways. These can be activated, inactivated, or switched by substrate cleavage (11).

• Activation can occur when ligand binding induces ectodomain shedding, enabling γ-secretase to cleave the remaining CTF and release an ICD that initiates downstream signaling, as first found for Notch (210). However, ICD-dependent signaling can also occur without prior ligand engagement, as shown for *e.g.*, LRP1 (211) and EPHA4 (212). For many substrates, their ICD translocates to the nucleus, where it can stimulate gene expression, as exemplified for Notch (210) and CD44 (213). γ-Secretase can also activate signaling when liberated ICDs release bound transcriptional regulators, as observed for CDH1 (214). Here, the transcriptional regulator β-catenin is sequestered at the CDH1 ICD. Upon γ-secretase cleavage of CDH1, β-catenin is released from the CDH1 ICD. This enables the nuclear translocation of β-catenin and activation of Wnt signaling. In other cases, liberated ICDs activate cytoplasmic signaling cascades, such as Rac GTPase signaling upon EPHA4 cleavage (212).
• Inactivation can occur when released ICDs translocate to the nucleus and repress gene expression (*e.g.*, for IFNAR2 (215)), or when they bind to cytosolic transcription factors and promote their subsequent degradation thereby preventing their nuclear import as in the case of CDH2 (216). Alternatively, when the intact CTF stimulates signaling, such as that for DCC *via* cAMP formation (217), γ-secretase cleavage of the CTF turns off the pathway.
• Switching between signaling pathways can occur when the full-length protein and the ICD are involved in different pathways, such that CTF cleavage by γ-secretase redirects signaling, as shown for CD44 (218), TNFRSF1A (219) or for some RTKs (220, 221). For instance, beyond canonical signaling by dimerized full-length RTKs (222), when RTK CTFs are cleaved by γ-secretase, their released ICDs have been implicated in gene expression, such as for ERBB4 and RYK (221) or cell proliferation in the case of AXL and TYRO3 (223).

The impact of γ-secretase cleavage is often multi-faceted. This functional complexity is, for example, illustrated by certain cell adhesion proteins for which γ-secretase cleavage contributes to their overall turnover as well as to actin cytoskeleton dynamics. This has been demonstrated for CDH1 (214) and EFNB1 (224), where the substrate ICD binds to the actin cytoskeleton and CTF cleavage by γ-secretase disrupts this interaction. Another layer of γ-secretase’s complex role in signaling is demonstrated by the functional consequences of its role in TREM2 turnover. TREM2 is involved in SYK signaling without γ-secretase-mediated liberation of its ICD. Instead, SYK signaling is triggered after ligand binding to a complex formed by full-length TREM2 and DAP12. When γ-secretase cleavage is impaired, accumulating TREM2 CTFs trap DAP12, reducing its interaction with full-length TREM2, thereby attenuating SYK signaling (225). Overall, the mechanisms by which γ-secretase regulates cellular processes are manifold, often complex, and highly context-dependent.

### The functional scope of γ-secretase across biological systems

γ-Secretase is critical in health and disease, spanning major biological systems—particularly the nervous, immune, and cardiovascular systems—where important associations can be described regarding biological processes/organs, cell types, and diseases (Table 3). Several comprehensive reviews have highlighted established substrate groups that define the broad functional spectrum of γ-secretase, most notably Notch receptors and ligands (210, 226, 227), the APP family (228, 229), RTKs (221), lipoprotein receptors (211), and cadherins (230). Notch receptors and ligands regulate cell fate and tissue homeostasis during development (210). The APP family, which besides APP includes APLP1 and APLP2, is involved in several neuronal processes including synapse formation and synapse function, with APP and APLP2 playing a particularly important role (228). RTKs—particularly ERBB4—connect γ-secretase processing to angiogenesis (231) and epithelial cell differentiation in, *e.g.*, mammary gland development (221). Lipoprotein receptors—notably LDLR and LRP1—link substrate cleavage to cholesterol metabolism (211), while LRP2 further ties γ-secretase to urinary reabsorption (232). Cadherins form another major substrate group. CDH1 is associated with epithelial integrity (214) and mammary glands (233); CDH2 with synaptic transmission (234) and CDH5 with angiogenesis (235) and endothelial permeability (236).

Beyond these families, numerous other substrates connect γ-secretase processing to more specialized, system-specific biological processes at the level of organs and cell types. In the nervous system, adhesion proteins dominate: the cadherin subfamily of protocadherins (PCDHA4 (237), PCDH17) (238)), nectins (NECTIN1, NECTIN3) (239), and neurexins (NRXN1, NRXN2) (240) link γ-secretase to synaptic development, synapse formation, and synapse function. Additional functions involve receptor-type tyrosine phosphatases (PTPRD, PTPRF) in axon guidance (241), sortilin (SORT1) and other VPS10 domain-containing receptors (SORCS1, SORL1) in neuronal trafficking (242), and NGFR in neuronal survival (243). Ion channel subunits form another key group: sodium channel β-subunits (SCN2B) influence neuronal excitability (244), while potassium channel β-subunits (KCNE1) couple cleavage with cardiomyocyte functions in the cardiovascular system (245). Immune-related functions are reflected by chemokines (CX3CL1, CXCL16) (246) and the inflammatory cytokine receptors IL1R1 (247) and IL1R2 (248). Other examples include SDC1 in wound healing (249), TYR and TYRP1 in pigmentation of melanocytes (250) as well as CD44, which is a versatile substrate (218, 251), functioning as a cell surface adhesion receptor associated with interferon responses (252), and also linked to cartilage and chondrocytes (253).

The disease spectrum of γ-secretase extends beyond its critical involvement in AD and Notch-driven cancers. In cancer, dysregulated γ-secretase substrate cleavage typically contributes either to loss of adhesion or to aberrant ICD signaling. Aberrant processing of AXL promotes melanoma (254) and lung cancer progression (255), CD44 cleavage alters adhesion and transcriptional programs in multiple cancer types such as colorectal (218), thyroid (256), and prostate cancers (257), and LRP8 processing links γ-secretase to gastric, prostate, breast, melanoma, and lung cancers (258). Additional examples include neuroblastoma (LRRN3 (259)), glioma (ERBB4 (260)), breast cancer (CDH1 (261), ERBB2 (262)), prostate cancer (DAG1 (263), TACSTD2 (264), CD44 (257)), and bladder cancer (CDH2 (265), FGFR3 (266)).

With regard to neurodegeneration, several examples highlight the disease relevance of γ-secretase and its substrates besides APP. Through its association with TREM2-dependent SYK signaling, γ-secretase contributes to microglial activity important for Aβ clearance in the brain (267). Lipoprotein receptors, especially LDLR and LRP1, are further implicated in APOE-related risk of AD (211). Substrates of γ-secretase and their processing are also implicated in other nervous system disorders, including DSCAM in Down syndrome (268), SCN1B in epilepsy (269), SCN2B additionally in multiple sclerosis (269), TREM2, besides AD, in frontotemporal dementia, Nasu-Hakola disease, and Parkinson’s disease (270), as well as ERBB4 with its ligand NRG1 in schizophrenia (271).

In addition, several γ-secretase-associated disorders share an immune or inflammatory component: atherosclerosis, where LDLR cleavage influences cholesterol uptake (272); diabetes, in which processing of APP (273) and CD44 (218) is linked to the metabolic homeostasis changes; pancreatitis, where aberrant JAG1/NOTCH1 signaling drives inflammation (210), and TNFRSF17 cleavage links γ-secretase to autoimmunity (34); and osteoarthritis, where CD44 processing impairs chondrocyte adhesion and extracellular matrix interactions, thereby promoting cartilage degeneration (253).

Altogether, this illustrates that γ-secretase substrates span almost all major biological systems in health and disease, with the neurovascular and immune compartments prominently represented. These findings highlight both the opportunities and risks of therapeutic strategies involving γ-secretase: while the broad substrate spectrum underlines the possibility of targeting γ-secretase for a variety of diseases, it also raises concerns about potential side effects, which must be mitigated through specific drug-targeting and careful monitoring.

## Outlook and open questions

As highlighted in this review, many mysteries of γ-secretase have been unraveled in the past few years, providing fascinating insights into the recognition and processing of its substrates, as well as the breadth of their scope. The entire substratome of γ-secretase—and thus its functional scope—has now been computationally predicted. Several new cellular pathways have been identified in which γ-secretase may play an important role, together with potential links to diseases beyond AD and cancer, stimulating further research to validate and elucidate these findings. In the course of these studies, it will also be interesting to determine whether there are differences between PS1 and PS2 in their physiological functions. What remains to be further explored is whether γ-secretase might also cleave polytopic membrane proteins, as previously indicated (36, 37, 38). If so, the functional scope of γ-secretase could be even broader than currently assumed.

Structural biology of γ-secretase has provided important insights into the sequential cleavage mechanism. However, because the underlying γ-secretase cryo-EM structures are static in nature, it will also be important to analyze the dynamics of the cleavage. We also do not yet know how FAD mutations in presenilin affect the structure of γ-secretase and the E–S complex. Similarly, it would be interesting to see if and how FAD mutations in the APP TMD alter the E–S complex. Such analyses would help to better understand the mechanism of pathogenic Aβ42/43 generation on a molecular level. Although the involvement of exosites in substrate recruitment has been established, the precise mechanism how the substrate gains access to the active site remains unresolved. In all E–S complex structures, the bound substrate is surrounded by a bundle of TMDs and protrudes laterally from the complex. However, hydrophilic loops in presenilin apparently present a barrier for the substrate to reach the active site. While hydrophilic loop 2 provides a barrier for the direct route between TMD2 and 3, the access route between TMD2 and 6 is laterally open to the substrate, but hydrophilic loop 1, which protrudes into the interior of the structure, must be passed and it is unclear how this occurs. But even if all this work continues to be a tour de force, if structural biology research on γ-secretase continues at the same pace as before, we will probably have answers to these questions within the next few years.

With regard to γ-secretase as a drug target, the concept of using GSMs as a therapeutic intervention for AD is very promising. Advanced clinical trials will hopefully show that the current clinical drug candidates are safe and effective, providing options for disease-modifying treatments of mild AD or even prevention of the disease. If so, GSMs could be used as monotherapy or in combination with anti-Aβ immunotherapies. Over 3 decades of intensive γ-secretase research would finally have paid off.

## Conflict of interest

The authors declare the following financial interests/personal relationships which may be considered as potential competing interests: H.S. has received research funding from Roche and speaker honorary from Astex Pharmaceuticals. All other authors declare that they have no conflicts of interest with the contents of this article.

## References

[1] Lopez-Otin C., Bond J.S. (2008). Proteases: multifunctional enzymes in life and disease. J. Biol. Chem..

[2] Erez E., Fass D., Bibi E. (2009). How intramembrane proteases bury hydrolytic reactions in the membrane. Nature.

[3] Sun L., Li X., Shi Y. (2016). Structural biology of intramembrane proteases: mechanistic insights from rhomboid and S2P to γ-secretase. Curr. Opin. Struct. Biol..

[4] Takasugi N., Tomita T., Hayashi I., Tsuruoka M., Niimura M., Takahashi Y. (2003). The role of presenilin cofactors in the γ-secretase complex. Nature.

[5] Edbauer D., Winkler E., Regula J.T., Pesold B., Steiner H., Haass C. (2003). Reconstitution of γ-secretase activity. Nat. Cell Biol..

[6] Kimberly W.T., LaVoie M.J., Ostaszewski B.L., Ye W., Wolfe M.S., Selkoe D.J. (2003). γ-Secretase is a membrane protein complex comprised of presenilin, nicastrin, Aph-1, and Pen-2. Proc. Natl. Acad. Sci. U. S. A..

[7] Steiner H., Fukumori A., Tagami S., Okochi M. (2018). Making the final cut: pathogenic amyloid-β peptide generation by γ-secretase. Cell Stress.

[8] Selkoe D.J., Hardy J. (2016). The amyloid hypothesis of Alzheimer's disease at 25 years. EMBO Mol. Med..

[9] Selkoe D.J. (2024). The advent of Alzheimer treatments will change the trajectory of human aging. Nat. Aging.

[10] De Strooper B., Karran E. (2024). New precision medicine avenues to the prevention of Alzheimer's disease from insights into the structure and function of γ-secretases. EMBO J..

[11] Jurisch-Yaksi N., Sannerud R., Annaert W. (2013). A fast growing spectrum of biological functions of γ-secretase in development and disease. Biochim. Biophys. Acta.

[12] Güner G., Lichtenthaler S.F. (2020). The substrate repertoire of γ-secretase/presenilin. Semin. Cell Dev. Biol..

[13] Weng A.P., Ferrando A.A., Lee W., Morris J.P., Silverman L.B., Sanchez-Irizarry C. (2004). Activating mutations of NOTCH1 in human T cell acute lymphoblastic leukemia. Science.

[14] Lichtenthaler S.F., Haass C., Steiner H. (2011). Regulated intramembrane proteolysis - lessons from amyloid precursor protein processing. J. Neurochem..

[15] Lichtenthaler S.F. (2010). α-Secretase in Alzheimer's disease: molecular identity, regulation and therapeutic potential. J. Neurochem..

[16] Vassar R., Kuhn P.H., Haass C., Kennedy M.E., Rajendran L., Wong P.C. (2014). Function, therapeutic potential and cell biology of BACE proteases: current status and future prospects. J. Neurochem..

[17] Sastre M., Steiner H., Fuchs K., Capell A., Multhaup G., Condron M.M. (2001). Presenilin-dependent γ-secretase processing of β-amyloid precursor protein at a site corresponding to the S3 cleavage of Notch. EMBO Rep..

[18] Gu Y., Misonou H., Sato T., Dohmae N., Takio K., Ihara Y. (2001). Distinct intramembrane cleavage of the β-amyloid precursor protein family resembling γ-secretase-like cleavage of Notch. J. Biol. Chem..

[19] Yu C., Kim S.H., Ikeuchi T., Xu H., Gasparini L., Wang R. (2001). Characterization of a presenilin-mediated APP carboxyl terminal fragment CTFγ: evidence for distinct mechanisms involved in γ-secretase processing of the APP and Notch1 transmembrane domains. J. Biol. Chem..

[20] Weidemann A., Eggert S., Reinhard F.B., Vogel M., Paliga K., Baier G. (2002). A novel ε-cleavage within the transmembrane domain of the Alzheimer amyloid precursor protein demonstrates homology with Notch processing. Biochemistry.

[21] Qi-Takahara Y., Morishima-Kawashima M., Tanimura Y., Dolios G., Hirotani N., Horikoshi Y. (2005). Longer forms of amyloid β protein: implications for the mechanism of intramembrane cleavage by γ-secretase. J. Neurosci..

[22] Zhao G., Cui M.Z., Mao G., Dong Y., Tan J., Sun L. (2005). γ-Cleavage is dependent on ζ-cleavage during the proteolytic processing of amyloid precursor protein within its transmembrane domain. J. Biol. Chem..

[23] Takami M., Nagashima Y., Sano Y., Ishihara S., Morishima-Kawashima M., Funamoto S. (2009). γ-Secretase: successive tripeptide and tetrapeptide release from the transmembrane domain of β-carboxyl terminal fragment. J. Neurosci..

[24] Iwatsubo T., Odaka A., Suzuki N., Mizusawa H., Nukina N., Ihara Y. (1994). Visualization of Aβ42(43) and Aβ40 in senile plaques with end-specific Aβ monoclonals: evidence that an initially deposited species is Aβ42(43). Neuron.

[25] Iizuka T., Shoji M., Harigaya Y., Kawarabayashi T., Watanabe M., Kanai M. (1995). Amyloid β-protein ending at Thr43 is a minor component of some diffuse plaques in the Alzheimer's disease brain, but is not found in cerebrovascular amyloid. Brain Res..

[26] Okochi M., Tagami S., Yanagida K., Takami M., Kodama T.S., Mori K. (2013). γ-Secretase modulators and presenilin 1 mutants act differently on presenilin/γ-secretase function to cleave Aβ42 and Aβ43. Cell Rep..

[27] Olsson F., Schmidt S., Althoff V., Munter L.M., Jin S., Rosqvist S. (2014). Characterization of intermediate steps in amyloid β (Aβ) production under near-native conditions. J. Biol. Chem..

[28] Okochi M., Steiner H., Fukumori A., Tanii H., Tomita T., Tanaka T. (2002). Presenilins mediate a dual intramembraneous γ-secretase cleavage of Notch-1. EMBO J..

[29] Lammich S., Okochi M., Takeda M., Kaether C., Capell A., Zimmer A.K. (2002). Presenilin-dependent intramembrane proteolysis of CD44 leads to the liberation of its intracellular domain and the secretion of an Aβ-like peptide. J. Biol. Chem..

[30] Yanagida K., Okochi M., Tagami S., Nakayama T., Kodama T.S., Nishitomi K. (2009). The 28-amino acid form of an APLP1-derived Aβ-like peptide is a surrogate marker for Aβ42 production in the central nervous system. EMBO Mol. Med..

[31] Ran Y., Hossain F., Pannuti A., Lessard C.B., Ladd G.Z., Jung J.I. (2017). γ-Secretase inhibitors in cancer clinical trials are pharmacologically and functionally distinct. EMBO Mol. Med..

[32] Kopan R., Ilagan M.X. (2004). γ-Secretase: proteasome of the membrane?. Nat. Rev. Mol. Cell Biol..

[33] Schroeter E.H., Kisslinger J.A., Kopan R. (1998). Notch-1 signalling requires ligand-induced proteolytic release of intracellular domain. Nature.

[34] Laurent S.A., Hoffmann F.S., Kuhn P.H., Cheng Q., Chu Y., Schmidt-Supprian M. (2015). γ-Secretase directly sheds the survival receptor BCMA from plasma cells. Nat. Commun..

[35] Güner G., Assfalg M., Zhao K., Dreyer T., Lahiri S., Lo Y. (2022). Proteolytically generated soluble tweak receptor Fn14 is a blood biomarker for γ-secretase activity. EMBO Mol. Med..

[36] Meyer E.L., Strutz N., Gahring L.C., Rogers S.W. (2003). Glutamate receptor subunit 3 is modified by site-specific limited proteolysis including cleavage by γ-secretase. J. Biol. Chem..

[37] Merrick D., Chapin H., Baggs J.E., Yu Z., Somlo S., Sun Z. (2012). The γ-secretase cleavage product of polycystin-1 regulates TCF and CHOP-mediated transcriptional activation through a p300-dependent mechanism. Dev. Cell.

[38] Restrepo L.J., DePew A.T., Moese E.R., Tymanskyj S.R., Parisi M.J., Aimino M.A. (2022). γ-Secretase promotes *Drosophila* postsynaptic development through the cleavage of a Wnt receptor. Dev. Cell.

[39] Erez E., Bibi E. (2009). Cleavage of a multispanning membrane protein by an intramembrane serine protease. Biochemistry.

[40] Fleig L., Bergbold N., Sahasrabudhe P., Geiger B., Kaltak L., Lemberg M.K. (2012). Ubiquitin-dependent intramembrane rhomboid protease promotes ERAD of membrane proteins. Mol. Cell.

[41] Wan C., Fu J., Wang Y., Miao S., Song W., Wang L. (2012). Exosome-related multi-pass transmembrane protein TSAP6 is a target of rhomboid protease RHBDD1-induced proteolysis. PLoS One.

[42] Began J., Cordier B., Brezinova J., Delisle J., Hexnerova R., Srb P. (2020). Rhomboid intramembrane protease YqgP licenses bacterial membrane protein quality control as adaptor of FtsH AAA protease. EMBO J..

[43] Grieve A.G., Yeh Y.C., Chang Y.F., Huang H.Y., Zarcone L., Breuning J. (2021). Conformational surveillance of Orai1 by a rhomboid intramembrane protease prevents inappropriate CRAC channel activation. Mol. Cell.

[44] Han S.I., Nakakuki M., Nakagawa Y., Wang Y., Araki M., Yamamoto Y. (2023). Rhomboid protease RHBDL4/RHBDD1 cleaves SREBP-1c at endoplasmic reticulum monitoring and regulating fatty acids. PNAS Nexus.

[45] Cao X., Südhof T.C. (2001). A transcriptionally active complex of APP with Fe65 and histone acetyltransferase Tip60. Science.

[46] Hebert S.S., Serneels L., Tolia A., Craessaerts K., Derks C., Filippov M.A. (2006). Regulated intramembrane proteolysis of amyloid precursor protein and regulation of expression of putative target genes. EMBO Rep..

[47] Aydin D., Filippov M.A., Tschape J.A., Gretz N., Prinz M., Eils R. (2011). Comparative transcriptome profiling of amyloid precursor protein family members in the adult cortex. BMC Genomics.

[48] Ring S., Weyer S.W., Kilian S.B., Waldron E., Pietrzik C.U., Filippov M.A. (2007). The secreted β-amyloid precursor protein ectodomain APPsα is sufficient to rescue the anatomical, behavioral, and electrophysiological abnormalities of APP-deficient mice. J. Neurosci..

[49] Kent S.A., Spires-Jones T.L., Durrant C.S. (2020). The physiological roles of tau and Aβ: implications for Alzheimer's disease pathology and therapeutics. Acta Neuropathol..

[50] DeKosky S.T., Scheff S.W. (1990). Synapse loss in frontal cortex biopsies in Alzheimer's disease: correlation with cognitive severity. Ann. Neurol..

[51] Terry R.D., Masliah E., Salmon D.P., Butters N., DeTeresa R., Hill R. (1991). Physical basis of cognitive alterations in Alzheimer's disease: synapse loss is the major correlate of cognitive impairment. Ann. Neurol..

[52] Puzzo D., Privitera L., Leznik E., Fa M., Staniszewski A., Palmeri A. (2008). Picomolar amyloid-β positively modulates synaptic plasticity and memory in hippocampus. J. Neurosci..

[53] Tzioras M., McGeachan R.I., Durrant C.S., Spires-Jones T.L. (2023). Synaptic degeneration in Alzheimer disease. Nat. Rev. Neurol..

[54] Lawrence J.L., Tong M., Alfulaij N., Sherrin T., Contarino M., White M.M. (2014). Regulation of presynaptic Ca^2+^, synaptic plasticity and contextual fear conditioning by a N-terminal β-amyloid fragment. J. Neurosci..

[55] Richter M.C., Ludewig S., Winschel A., Abel T., Bold C., Salzburger L.R. (2018). Distinct in vivo roles of secreted APP ectodomain variants APPsα and APPsβ in regulation of spine density, synaptic plasticity, and cognition. EMBO J..

[56] Beher D., Wrigley J.D., Owens A.P., Shearman M.S. (2002). Generation of C-terminally truncated amyloid-β peptides is dependent on γ-secretase activity. J. Neurochem..

[57] Portelius E., Price E., Brinkmalm G., Stiteler M., Olsson M., Persson R. (2011). A novel pathway for amyloid precursor protein processing. Neurobiol. Aging.

[58] Morrissey J.A., Mockett B.G., Singh A., Kweon D., Ohline S.M., Tate W.P. (2019). A C-terminal peptide from secreted amyloid precursor protein-α enhances long-term potentiation in rats and a transgenic mouse model of Alzheimer's disease. Neuropharmacology.

[59] Mockett B.G., Richter M., Abraham W.C., Müller U.C. (2017). Therapeutic potential of secreted amyloid precursor protein APPsα. Front. Mol. Neurosci..

[60] Rehra L., Erdinger S., Wagner L., Baltissen D., Just J., Konig L. (2026). Brain delivery of a neurotrophic peptide derived from secreted amyloid precursor protein APPsα as a therapeutic strategy for Alzheimer's disease. J. Control. Release.

[61] Thinakaran G., Borchelt D.R., Lee M.K., Slunt H.H., Spitzer L., Kim G. (1996). Endoproteolysis of presenilin 1 and accumulation of processed derivatives in vivo. Neuron.

[62] Podlisny M.B., Citron M., Amarante P., Sherrington R., Xia W., Zhang J. (1997). Presenilin proteins undergo heterogeneous endoproteolysis between Thr291 and Ala299 and occur as stable N- and C-terminal fragments in normal and Alzheimer brain tissue. Neurobiol. Dis..

[63] Wolfe M.S., Xia W., Ostaszewski B.L., Diehl T.S., Kimberly W.T., Selkoe D.J. (1999). Two transmembrane aspartates in presenilin-1 required for presenilin endoproteolysis and γ-secretase activity. Nature.

[64] Steiner H., Duff K., Capell A., Romig H., Grim M.G., Lincoln S. (1999). A loss of function mutation of presenilin-2 interferes with amyloid β-peptide production and Notch signaling. J. Biol. Chem..

[65] Beher D., Wrigley J.D., Nadin A., Evin G., Masters C.L., Harrison T. (2001). Pharmacological knock-down of the presenilin 1 heterodimer by a novel γ-secretase inhibitor: implications for presenilin biology. J. Biol. Chem..

[66] Fukumori A., Fluhrer R., Steiner H., Haass C. (2010). Three-amino acid spacing of presenilin endoproteolysis suggests a general stepwise cleavage of γ-secretase-mediated intramembrane proteolysis. J. Neurosci..

[67] De Strooper B., Saftig P., Craessaerts K., Vanderstichele H., Guhde G., Annaert W. (1998). Deficiency of presenilin-1 inhibits the normal cleavage of amyloid precursor protein. Nature.

[68] Naruse S., Thinakaran G., Luo J.J., Kusiak J.W., Tomita T., Iwatsubo T. (1998). Effects of PS1 deficiency on membrane protein trafficking in neurons. Neuron.

[69] Citron M., Westaway D., Xia W., Carlson G., Diehl T., Levesque G. (1997). Mutant presenilins of Alzheimer's disease increase production of 42-residue amyloid β-protein in both transfected cells and transgenic mice. Nat. Med..

[70] Wolfe M.S., Citron M., Diehl T.S., Xia W., Donkor I.O., Selkoe D.J. (1998). A substrate-based difluoro ketone selectively inhibits Alzheimer's γ-secretase activity. J. Med. Chem..

[71] Wolfe M.S., Xia W., Moore C.L., Leatherwood D.D., Ostaszewski B., Rahmati T. (1999). Peptidomimetic probes and molecular modeling suggest that Alzheimer's γ-secretase is an intramembrane-cleaving aspartyl protease. Biochemistry.

[72] Li Y.M., Lai M.T., Xu M., Huang Q., DiMuzio-Mower J., Sardana M.K. (2000). Presenilin 1 is linked with γ-secretase activity in the detergent solubilized state. Proc. Natl. Acad. Sci. U. S. A..

[73] Shearman M.S., Beher D., Clarke E.E., Lewis H.D., Harrison T., Hunt P. (2000). L-685,458, an aspartyl protease transition state mimic, is a potent inhibitor of amyloid β-protein precursor γ-secretase activity. Biochemistry.

[74] Kimberly W.T., Xia W., Rahmati T., Wolfe M.S., Selkoe D.J. (2000). The transmembrane aspartates in presenilin 1 and 2 are obligatory for γ-secretase activity and amyloid β-protein generation. J. Biol. Chem..

[75] Li Y.M., Xu M., Lai M.T., Huang Q., Castro J.L., DiMuzio-Mower J. (2000). Photoactivated γ-secretase inhibitors directed to the active site covalently label presenilin 1. Nature.

[76] Esler W.P., Kimberly W.T., Ostaszewski B.L., Diehl T.S., Moore C.L., Tsai J.-Y. (2000). Transition-state analogue inhibitors of γ-secretase bind directly to presenilin-1. Nat. Cell Biol..

[77] Seiffert D., Bradley J.D., Rominger C.M., Rominger D.H., Yang F., Meredith J.E. (2000). Presenilin-1 and -2 are molecular targets for γ-secretase inhibitors. J. Biol. Chem..

[78] Steiner H., Kostka M., Romig H., Basset G., Pesold B., Hardy J. (2000). Glycine 384 is required for presenilin-1 function and is conserved in polytopic bacterial aspartyl proteases. Nat. Cell Biol..

[79] Weihofen A., Binns K., Lemberg M.K., Ashman K., Martoglio B. (2002). Identification of signal peptide peptidase, a presenilin-type aspartic protease. Science.

[80] Ponting C.P., Hutton M., Nyborg A., Baker M., Jansen K., Golde T.E. (2002). Identification of a novel family of presenilin homologues. Hum. Mol. Genet..

[81] Grigorenko A.P., Moliaka Y.K., Korovaitseva G.I., Rogaev E.I. (2002). Novel class of polytopic proteins with domains associated with putative protease activity. Biochemistry (Mosc).

[82] Fraering P.C., LaVoie M.J., Ye W., Ostaszewski B.L., Kimberly W.T., Selkoe D.J. (2004). Detergent-dependent dissociation of active γ-secretase reveals an interaction between Pen-2 and PS1-NTF and offers a model for subunit organization within the complex. Biochemistry.

[83] Steiner H., Winkler E., Haass C. (2008). Chemical cross-linking provides a model of the γ-secretase complex subunit architecture and evidence for close proximity of the C-terminal fragment of presenilin with APH-1. J. Biol. Chem..

[84] Sun L., Zhao L., Yang G., Yan C., Zhou R., Zhou X. (2015). Structural basis of human γ-secretase assembly. Proc. Natl. Acad. Sci. U. S. A..

[85] Bai X.C., Yan C., Yang G., Lu P., Ma D., Sun L. (2015). An atomic structure of human γ-secretase. Nature.

[86] Guo X., Wang Y., Zhou J., Jin C., Wang J., Jia B. (2022). Molecular basis for isoform-selective inhibition of presenilin-1 by MRK-560. Nat. Commun..

[87] Luo W.J., Wang H., Li H., Kim B.S., Shah S., Lee H.J. (2003). PEN-2 and APH-1 coordinately regulate proteolytic processing of presenilin 1. J. Biol. Chem..

[88] Kim S.H., Yin Y.I., Li Y.M., Sisodia S.S. (2004). Evidence that assembly of an active γ-secretase complex occurs in the early compartments of the secretory pathway. J. Biol. Chem..

[89] Capell A., Beher D., Prokop S., Steiner H., Kaether C., Shearman M.S. (2005). γ-Secretase complex assembly within the early secretory pathway. J. Biol. Chem..

[90] Kim J., Kleizen B., Choy R., Thinakaran G., Sisodia S.S., Schekman R.W. (2007). Biogenesis of γ-secretase early in the secretory pathway. J. Cell Biol..

[91] Wouters R., Michiels C., Sannerud R., Kleizen B., Dillen K., Vermeire W. (2021). Assembly of γ-secretase occurs through stable dimers after exit from the endoplasmic reticulum. J. Cell Biol..

[92] Meckler X., Checler F. (2016). Presenilin 1 and presenilin 2 target γ-secretase complexes to distinct cellular compartments. J. Biol. Chem..

[93] Sannerud R., Esselens C., Ejsmont P., Mattera R., Rochin L., Tharkeshwar A.K. (2016). Restricted location of PSEN2/γ-secretase determines substrate specificity and generates an intracellular Aβ pool. Cell.

[94] Kinoshita J., Strobel G., Jucker M., Beyreuther K., Haass C., Nitsch R.M., Christen Y. (2006). Alzheimer Research Forum: A Knowledge Base and e-community for AD Research in Alzheimer: 100 Years and Beyond.

[95] Alzforum (n.d.) Mutations. Alzforum (Retrieved January 26, 2026).

[96] Chavez-Gutierrez L., Bammens L., Benilova I., Vandersteen A., Benurwar M., Borgers M. (2012). The mechanism of γ-secretase dysfunction in familial Alzheimer disease. EMBO J..

[97] Scheuner D., Eckman C., Jensen M., Song X., Citron M., Suzuki N. (1996). Secreted amyloid β-protein similar to that in the senile plaques of Alzheimer's disease is increased in vivo by the presenilin 1 and 2 and APP mutations linked to familial Alzheimer's disease. Nat. Med..

[98] Saito T., Suemoto T., Brouwers N., Sleegers K., Funamoto S., Mihira N. (2011). Potent amyloidogenicity and pathogenicity of Aβ43. Nat. Neurosci..

[99] Veugelen S., Saito T., Saido T.C., Chavez-Gutierrez L., De Strooper B. (2016). Familial Alzheimer’s disease mutations in presenilin generate amyloidogenic Aβ peptide seeds. Neuron.

[100] Kretner B., Trambauer J., Fukumori A., Mielke J., Kuhn P.H., Kremmer E. (2016). Generation and deposition of Aβ43 by the virtually inactive presenilin-1 L435F mutant contradicts the presenilin loss-of-function hypothesis of Alzheimer's disease. EMBO Mol. Med..

[101] Petit D., Fernandez S.G., Zoltowska K.M., Enzlein T., Ryan N.S., O'Connor A. (2022). Aβ profiles generated by Alzheimer's disease causing PSEN1 variants determine the pathogenicity of the mutation and predict age at disease onset. Mol. Psychiatry.

[102] Fernandez S.G., Oria C.G., Petit D., Annaert W., Ringman J.M., Fox N.C. (2025). Spectrum of γ-secretase dysfunction as a unifying predictor of ADAD age at onset across PSEN1, PSEN2 and APP causal genes. Mol. Neurodegener..

[103] Quintero-Monzon O., Martin M.M., Fernandez M.A., Cappello C.A., Krzysiak A.J., Osenkowski P. (2011). Dissociation between the processivity and total activity of γ-secretase: implications for the mechanism of Alzheimer's disease-causing presenilin mutations. Biochemistry.

[104] Devkota S., Williams T.D., Wolfe M.S. (2021). Familial Alzheimer's disease mutations in amyloid protein precursor alter proteolysis by γ-secretase to increase amyloid β-peptides of ≥ 45 residues. J. Biol. Chem..

[105] Szaruga M., Munteanu B., Lismont S., Veugelen S., Horre K., Mercken M. (2017). Alzheimer's-causing mutations shift Aβ length by destabilizing γ-secretase-Aβn Interactions. Cell.

[106] Van Vickle G.D., Esh C.L., Kokjohn T.A., Patton R.L., Kalback W.M., Luehrs D.C. (2008). Presenilin-1 280Glu→Ala mutation alters C-terminal APP processing yielding longer Aβ peptides: implications for Alzheimer's disease. Mol. Med..

[107] Tang N., Kepp K.P. (2018). Aβ42/Aβ40 ratios of presenilin 1 mutations correlate with clinical onset of Alzheimer's disease. J. Alzheimers Dis..

[108] Liu L., Lauro B.M., He A., Lee H., Bhattarai S., Wolfe M.S. (2023). Identification of the Aβ37/42 peptide ratio in CSF as an improved Aβ biomarker for Alzheimer's disease. Alzheimers Dement..

[109] Schultz S.A., Liu L., Schultz A.P., Fitzpatrick C.D., Levin R., Bellier J.P. (2024). γ-Secretase activity, clinical features, and biomarkers of autosomal dominant Alzheimer's disease: cross-sectional and longitudinal analysis of the dominantly inherited Alzheimer network observational study (DIAN-OBS). Lancet Neurol..

[110] Fukumori A., Steiner H. (2016). Substrate recruitment of γ-secretase and mechanism of clinical presenilin mutations revealed by photoaffinity mapping. EMBO J..

[111] Trambauer J., Rodriguez Sarmiento R.M., Fukumori A., Feederle R., Baumann K., Steiner H. (2020). Aβ43-producing PS1 FAD mutants cause altered substrate interactions and respond to γ-secretase modulation. EMBO Rep..

[112] Mehra R., Kepp K.P. (2019). Computational analysis of Alzheimer-causing mutations in amyloid precursor protein and presenilin 1. Arch. Biochem. Biophys..

[113] Devkota S., Zhou R., Nagarajan V., Maesako M., Do H., Noorani A. (2024). Familial Alzheimer mutations stabilize synaptotoxic γ-secretase-substrate complexes. Cell Rep..

[114] Arafi P., Devkota S., Williams E., Maesako M., Wolfe M.S. (2025). Alzheimer-mutant γ-secretase complexes stall amyloid β-peptide production. Elife.

[115] Wolfe M.S. (2025). Presenilin, γ-secretase, and the search for pathogenic triggers of Alzheimer's disease. Biochemistry.

[116] Maruyama R., Fukumori A., Funamoto S., Okada K., Akamine S., Yanagida K. (2026). γ-Secretase exosites as targets for substrate-selective lowering of Aβ generation. Structure.

[117] Funamoto S., Sasaki T., Ishihara S., Nobuhara M., Nakano M., Watanabe-Takahashi M. (2013). Substrate ectodomain is critical for substrate preference and inhibition of γ-secretase. Nat. Commun..

[118] Tian G., Sobotka-Briner C.D., Zysk J., Liu X., Birr C., Sylvester M.A. (2002). Linear non-competitive inhibition of solubilized human γ-secretase by pepstatin A methylester, L685458, sulfonamides, and benzodiazepines. J. Biol. Chem..

[119] Esler W.P., Kimberly W.T., Ostaszewski B.L., Ye W., Diehl T.S., Selkoe D.J. (2002). Activity-dependent isolation of the presenilin–γ-secretase complex reveals nicastrin and a γ substrate. Proc. Natl. Acad. Sci. U. S. A..

[120] Kornilova A.Y., Bihel F., Das C., Wolfe M.S. (2005). The initial substrate-binding site of γ-secretase is located on presenilin near the active site. Proc. Natl. Acad. Sci. U. S. A..

[121] Lu P., Bai X.C., Ma D., Xie T., Yan C., Sun L. (2014). Three-dimensional structure of human γ-secretase. Nature.

[122] Zhou R., Yang G., Guo X., Zhou Q., Lei J., Shi Y. (2019). Recognition of the amyloid precursor protein by human γ-secretase. Science.

[123] Yang G., Zhou R., Zhou Q., Guo X., Yan C., Ke M. (2019). Structural basis of Notch recognition by human γ-secretase. Nature.

[124] Chen S.Y., Feilen L.P., Chavez-Gutierrez L., Steiner H., Zacharias M. (2023). Enzyme-substrate hybrid β-sheet controls geometry and water access to the γ-secretase active site. Commun. Biol..

[125] Bhattarai A., Devkota S., Bhattarai S., Wolfe M.S., Miao Y. (2020). Mechanisms of γ-secretase activation and substrate processing. ACS Cent. Sci..

[126] Bhattarai A., Devkota S., Do H.N., Wang J., Bhattarai S., Wolfe M.S. (2022). Mechanism of tripeptide trimming of amyloid β-peptide 49 by γ-secretase. J. Am. Chem. Soc..

[127] Guo X., Li H., Yan C., Lei J., Zhou R., Shi Y. (2024). Molecular mechanism of substrate recognition and cleavage by human γ-secretase. Science.

[128] Odorcic I., Hamed M.B., Lismont S., Chavez-Gutierrez L., Efremov R.G. (2024). Apo and Aβ46-bound γ-secretase structures provide insights into amyloid-β processing by the APH-1B isoform. Nat. Commun..

[129] Yang G., Zhou R., Guo X., Yan C., Lei J., Shi Y. (2021). Structural basis of γ-secretase inhibition and modulation by small molecule drugs. Cell.

[130] Tyndall J.D., Nall T., Fairlie D.P. (2005). Proteases universally recognize β strands in their active sites. Chem. Rev..

[131] Zoll S., Stanchev S., Began J., Skerle J., Lepsik M., Peclinovska L. (2014). Substrate binding and specificity of rhomboid intramembrane protease revealed by substrate-peptide complex structures. EMBO J..

[132] Imaizumi Y., Takanuki K., Miyake T., Takemoto M., Hirata K., Hirose M. (2022). Mechanistic insights into intramembrane proteolysis by *E. coli* site-2 protease homolog RseP. Sci. Adv..

[133] Orlando M.A., Pouillon H.J.T., Mandal S., Kroos L., Orlando B.J. (2024). Substrate engagement by the intramembrane metalloprotease SpoIVFB. Nat. Commun..

[134] Ren Z., Schenk D., Basi G.S., Shapiro I.P. (2007). Amyloid β-protein precursor juxtamembrane domain regulates specificity of γ-secretase-dependent cleavages. J. Biol. Chem..

[135] Page R.M., Gutsmiedl A., Fukumori A., Winkler E., Haass C., Steiner H. (2010). β-Amyloid precursor protein mutants respond to γ-secretase modulators. J. Biol. Chem..

[136] Kukar T.L., Ladd T.B., Robertson P., Pintchovski S.A., Moore B., Bann M.A. (2011). Lysine 624 of the amyloid precursor protein (APP) is a critical determinant of amyloid β peptide length: support for a sequential model of γ-secretase intramembrane proteolysis and regulation by the amyloid β precursor protein (APP) juxtamembrane region. J. Biol. Chem..

[137] Ousson S., Saric A., Baguet A., Losberger C., Genoud S., Vilbois F. (2013). Substrate determinants in the C99 juxtamembrane domains differentially affect γ-secretase cleavage specificity and modulator pharmacology. J. Neurochem..

[138] Jung J.I., Ran Y., Cruz P.E., Rosario A.M., Ladd T.B., Kukar T.L. (2014). Complex relationships between substrate sequence and sensitivity to alterations in γ-secretase processivity induced by γ-secretase modulators. Biochemistry.

[139] Petit D., Hitzenberger M., Lismont S., Zoltowska K.M., Ryan N.S., Mercken M. (2019). Extracellular interface between APP and nicastrin regulates Aβ length and response to γ-secretase modulators. EMBO J..

[140] Koch M., Enzlein T., Chen S.Y., Petit D., Lismont S., Zacharias M. (2023). APP substrate ectodomain defines amyloid-β peptide length by restraining γ-secretase processivity and facilitating product release. EMBO J..

[141] Jakowiecki J., Orzel U., Miszta P., Mlynarczyk K., Filipek S. (2024). Conformational changes and unfolding of β-amyloid substrates in the active site of γ-secretase. Int. J. Mol. Sci..

[142] Fernandez M.A., Biette K.M., Dolios G., Seth D., Wang R., Wolfe M.S. (2016). Transmembrane substrate determinants for γ-secretase processing of APP CTFβ. Biochemistry.

[143] Zhou H., Zhou S., Walian P.J., Jap B.K. (2010). Dependency of γ-secretase complex activity on the structural integrity of the bilayer. Biochem. Biophys. Res. Commun..

[144] Feilen L.P., Chen S.Y., Fukumori A., Feederle R., Zacharias M., Steiner H. (2022). Active site geometry stabilization of a presenilin homolog by the lipid bilayer promotes intramembrane proteolysis. Elife.

[145] Osenkowski P., Ye W., Wang R., Wolfe M.S., Selkoe D.J. (2008). Direct and potent regulation of γ-secretase by its lipid microenvironment. J. Biol. Chem..

[146] Holmes O., Paturi S., Ye W., Wolfe M.S., Selkoe D.J. (2012). Effects of membrane lipids on the activity and processivity of purified γ-secretase. Biochemistry.

[147] Onodera T., Futai E., Kan E., Abe N., Uchida T., Kamio Y. (2015). Phosphatidylethanolamine plasmalogen enhances the inhibiting effect of phosphatidylethanolamine on γ-secretase activity. J. Biochem..

[148] Aguayo-Ortiz R., Straub J.E., Dominguez L. (2018). Influence of membrane lipid composition on the structure and activity of γ-secretase. Phys. Chem. Chem. Phys..

[149] Winkler E., Kamp F., Scheuring J., Ebke A., Fukumori A., Steiner H. (2012). Generation of Alzheimer disease-associated amyloid β42/43 peptide by γ-secretase can be inhibited directly by modulation of membrane thickness. J. Biol. Chem..

[150] Ayciriex S., Gerber H., Osuna G.M., Chami M., Stahlberg H., Shevchenko A. (2016). The lipidome associated with the γ-secretase complex is required for its integrity and activity. Biochem. J..

[151] Dawkins E., Derks R.J.E., Schifferer M., Trambauer J., Winkler E., Simons M. (2023). Membrane lipid remodeling modulates γ-secretase processivity. J. Biol. Chem..

[152] Kong L., Dawkins E., Campbell F., Winkler E., Derks R.J.E., Giera M. (2020). Photo-controlled delivery of very long chain fatty acids to cell membranes and modulation of membrane protein function. Biochim. Biophys. Acta Biomembr..

[153] Colombo A.V., Sadler R.K., Llovera G., Singh V., Roth S., Heindl S. (2021). Microbiota-derived short chain fatty acids modulate microglia and promote Aβ plaque deposition. Elife.

[154] Wang X., Zhou R., Sun X., Li J., Wang J., Yue W. (2023). Preferential regulation of γ-secretase-mediated cleavage of APP by ganglioside GM1 reveals a potential therapeutic target for Alzheimer's disease. Adv. Sci. (Weinh).

[155] Jung J.I., Ladd T.B., Kukar T., Price A.R., Moore B.D., Koo E.H. (2013). Steroids as γ-secretase modulators. FASEB J..

[156] Jung J.I., Price A.R., Ladd T.B., Ran Y., Park H.J., Ceballos-Diaz C. (2015). Cholestenoic acid, an endogenous cholesterol metabolite, is a potent γ-secretase modulator. Mol. Neurodegener..

[157] Doody R.S., Raman R., Farlow M., Iwatsubo T., Vellas B., Joffe S. (2013). A phase 3 trial of semagacestat for treatment of Alzheimer's disease. N. Engl. J. Med..

[158] Coric V., Salloway S., van Dyck C.H., Dubois B., Andreasen N., Brody M. (2015). Targeting prodromal Alzheimer disease with avagacestat: a randomized clinical trial. JAMA Neurol..

[159] Tagami S., Yanagida K., Kodama T.S., Takami M., Mizuta N., Oyama H. (2017). Semagacestat is a pseudo-inhibitor of γ-secretase. Cell Rep..

[160] Kwart D., Gregg A., Scheckel C., Murphy E., Paquet D., Duffield M. (2019). A large panel of isogenic APP and PSEN1 mutant human iPSC neurons reveals shared endosomal abnormalities mediated by APP β-CTFs, not Aβ. Neuron.

[161] Bretou M., Sannerud R., Escamilla-Ayala A., Leroy T., Vrancx C., Van Acker Z.P. (2024). Accumulation of APP C-terminal fragments causes endolysosomal dysfunction through the dysregulation of late endosome to lysosome-ER contact sites. Dev. Cell.

[162] Habets R.A., de Bock C.E., Serneels L., Lodewijckx I., Verbeke D., Nittner D. (2019). Safe targeting of T cell acute lymphoblastic leukemia by pathology-specific NOTCH inhibition. Sci. Transl. Med..

[163] Gounder M., Ratan R., Alcindor T., Schoffski P., van der Graaf W.T., Wilky B.A. (2023). Nirogacestat, a γ-secretase inhibitor for desmoid tumors. N. Engl. J. Med..

[164] Weggen S., Eriksen J.L., Das P., Sagi S.A., Wang R., Pietrzik C.U. (2001). A subset of NSAIDs lower amyloidogenic Aβ42 independently of cyclooxygenase activity. Nature.

[165] Weggen S., Eriksen J.L., Sagi S.A., Pietrzik C.U., Golde T.E., Koo E.H. (2003). Aβ42-lowering nonsteroidal anti-inflammatory drugs preserve intramembrane cleavage of the amyloid precursor protein (APP) and ErbB-4 receptor and signaling through the APP intracellular domain. J. Biol. Chem..

[166] Mekala S., Nelson G., Li Y.M. (2020). Recent developments of small molecule γ-secretase modulators for Alzheimer's disease. RSC Med. Chem..

[167] Kounnas M.Z., Danks A.M., Cheng S., Tyree C., Ackerman E., Zhang X. (2010). Modulation of γ-secretase reduces β-amyloid deposition in a transgenic mouse model of Alzheimer's disease. Neuron.

[168] Ebke A., Luebbers T., Fukumori A., Shirotani K., Haass C., Baumann K. (2011). Novel γ-secretase enzyme modulators directly target presenilin protein. J. Biol. Chem..

[169] Crump C.J., Fish B.A., Castro S.V., Chau D.M., Gertsik N., Ahn K. (2011). Piperidine acetic acid based γ-secretase modulators directly bind to presenilin-1. ACS Chem. Neurosci..

[170] Ohki Y., Higo T., Uemura K., Shimada N., Osawa S., Berezovska O. (2011). Phenylpiperidine-type γ-secretase modulators target the transmembrane domain 1 of presenilin 1. EMBO J..

[171] Jumpertz T., Rennhack A., Ness J., Baches S., Pietrzik C.U., Bulic B. (2012). Presenilin is the molecular target of acidic γ-secretase modulators in living cells. PLoS One.

[172] Pozdnyakov N., Murrey H.E., Crump C.J., Pettersson M., Ballard T.E., Am Ende C.W. (2013). γ-Secretase modulator (GSM) photoaffinity probes reveal distinct allosteric binding sites on presenilin. J. Biol. Chem..

[173] Takeo K., Tanimura S., Shinoda T., Osawa S., Zahariev I.K., Takegami N. (2014). Allosteric regulation of γ-secretase activity by a phenylimidazole-type γ-secretase modulator. Proc. Natl. Acad. Sci. U. S. A..

[174] Liu L., Lauro B.M., Wolfe M.S., Selkoe D.J. (2021). Hydrophilic loop 1 of Presenilin-1 and the APP GxxxG transmembrane motif regulate γ-secretase function in generating alzheimer-causing Aβ peptides. J. Biol. Chem..

[175] Petit D., Hitzenberger M., Koch M., Lismont S., Zoltowska K.M., Enzlein T. (2022). Enzyme-substrate interface targeting by imidazole-based γ-secretase modulators activates γ-secretase and stabilizes its interaction with APP. EMBO J..

[176] Lindemann L., Lambotte J., Rothe J., Messer J., Diener C., Pichereau S. (2026). Pharmacology of nivegacetor (RG6289), a potent and selective gamma secretase modulator, in clinical development for the treatment of Alzheimer’s disease. Front. Pharmacol..

[177] Mueggler T., Portrin A., Poirier A., Vogt A., Yang T., Abdi M.A. (2024). Pharmacodynamic effect of a new γ-secretase modulator, RG6289, on CSF amyloid-β peptides in a randomized phase I study. Alzheimers Dement..

[178] Fagan T. (2023).

[179] Quartey M.O., Nyarko J.N.K., Maley J.M., Barnes J.R., Bolanos M.A.C., Heistad R.M. (2021). The Aβ(1-38) peptide is a negative regulator of the Aβ(1-42) peptide implicated in Alzheimer disease progression. Sci. Rep..

[180] Braun G.A., Dear A.J., Sanagavarapu K., Zetterberg H., Linse S. (2022). Amyloid-β peptide 37, 38 and 40 individually and cooperatively inhibit amyloid-β 42 aggregation. Chem. Sci..

[181] Cullen N., Janelidze S., Palmqvist S., Stomrud E., Mattsson-Carlgren N., Hansson O. (2021). Association of CSF Aβ38 levels with risk of Alzheimer disease-related decline. Neurology.

[182] Szaruga M., Veugelen S., Benurwar M., Lismont S., Sepulveda-Falla D., Lleo A. (2015). Qualitative changes in human γ-secretase underlie familial Alzheimer's disease. J. Exp. Med..

[183] Trambauer J., Fukumori A., Steiner H. (2020). Pathogenic Aβ generation in familial Alzheimer's disease: novel mechanistic insights and therapeutic implications. Curr. Opin. Neurobiol..

[184] Trambauer J., Rodriguez Sarmiento R.M., Garringer H.J., Salbaum K., Pedro L.P., Crusius D. (2025). γ-Secretase modulator resistance of an aggressive Alzheimer-causing presenilin mutant can be overcome in the heterozygous patient state by a set of advanced compounds. Alz. Res. Ther..

[185] Bright A. (2025).

[186] Okochi M., Fukumori A., Jiang J., Itoh N., Kimura R., Steiner H. (2006). Secretion of the Notch-1 Aβ-like peptide during Notch signaling. J. Biol. Chem..

[187] Wanngren J., Ottervald J., Parpal S., Portelius E., Stromberg K., Borgegard T. (2012). Second generation γ-secretase modulators exhibit different modulation of Notch β and Aβ production. J. Biol. Chem..

[188] Hemming M.L., Elias J.E., Gygi S.P., Selkoe D.J. (2008). Proteomic profiling of γ-secretase substrates and mapping of substrate requirements. PLoS Biol..

[189] Aßfalg M., Güner G., Müller S.A., Breimann S., Langosch D., Muhle-Goll C. (2024). Cleavage efficiency of the intramembrane protease γ-secretase is reduced by the palmitoylation of a substrate's transmembrane domain. FASEB J..

[190] Pester O., Barrett P.J., Hornburg D., Hornburg P., Probstle R., Widmaier S. (2013). The backbone dynamics of the amyloid precursor protein transmembrane helix provides a rationale for the sequential cleavage mechanism of γ-secretase. J. Am. Chem. Soc..

[191] Götz A., Mylonas N., Hogel P., Silber M., Heinel H., Menig S. (2019). Modulating Hinge flexibility in the APP transmembrane domain alters γ-secretase cleavage. Biophys. J..

[192] Ortner M., Guschtschin-Schmidt N., Stelzer W., Muhle-Goll C., Langosch D. (2023). Permissive conformations of a transmembrane helix allow intramembrane proteolysis by γ-secretase. J. Mol. Biol..

[193] Werner N.T., Hogel P., Guner G., Stelzer W., Wozny M., Assfalg M. (2023). Cooperation of N- and C-terminal substrate transmembrane domain segments in intramembrane proteolysis by γ-secretase. Commun. Biol..

[194] Steiner A., Schlepckow K., Brunner B., Steiner H., Haass C., Hagn F. (2020). γ-Secretase cleavage of the Alzheimer risk factor TREM2 is determined by its intrinsic structural dynamics. EMBO J..

[195] Kamp F., Winkler E., Trambauer J., Ebke A., Fluhrer R., Steiner H. (2015). Intramembrane proteolysis of β-amyloid precursor protein by γ-secretase is an unusually slow process. Biophys. J..

[196] Bolduc D.M., Montagna D.R., Gu Y., Selkoe D.J., Wolfe M.S. (2016). Nicastrin functions to sterically hinder γ-secretase–substrate interactions driven by substrate transmembrane domain. Proc. Natl. Acad. Sci. U. S. A..

[197] Araya L.E., Soni I.V., Hardy J.A., Julien O. (2021). Deorphanizing caspase-3 and caspase-9 substrates in and out of apoptosis with deep substrate profiling. ACS Chem. Biol..

[198] Beel A.J., Sanders C.R. (2008). Substrate specificity of γ-secretase and other intramembrane proteases. Cell. Mol. Life Sci..

[199] Strisovsky K., Sharpe H.J., Freeman M. (2009). Sequence-specific intramembrane proteolysis: identification of a recognition motif in rhomboid substrates. Mol. Cell.

[200] Moin S.M., Urban S. (2012). Membrane immersion allows rhomboid proteases to achieve specificity by reading transmembrane segment dynamics. Elife.

[201] Yücel S.S., Stelzer W., Lorenzoni A., Wozny M., Langosch D., Lemberg M.K. (2019). The metastable XBP1u transmembrane domain defines determinants for intramembrane proteolysis by signal peptide peptidase. Cell Rep..

[202] Breimann S., Kamp F., Basset G., Abou-Ajram C., Güner G., Yanagida K. (2025). Charting γ-secretase substrates by explainable AI. Nat. Commun..

[203] Langosch D., Steiner H. (2017). Substrate processing in intramembrane proteolysis by γ-secretase - the role of protein dynamics. Biol. Chem..

[204] Li F., Wang C., Guo X., Akutsu T., Webb G.I., Coin L.J.M. (2023). ProsperousPlus: a one-stop and comprehensive platform for accurate protease-specific substrate cleavage prediction and machine-learning model construction. Brief. Bioinform..

[205] Breimann S., Kamp F., Steiner H., Frishman D. (2024). AAontology: an ontology of amino acid scales for interpretable machine learning. J. Mol. Biol..

[206] Killian J.A., von Heijne G. (2000). How proteins adapt to a membrane-water interface. Trends Biochem. Sci..

[207] Lundberg S.M., Erion G., Chen H., DeGrave A., Prutkin J.M., Nair B. (2020). From local explanations to global understanding with explainable AI for trees. Nat. Mach. Intell..

[208] Hou P., Zielonka M., Serneels L., Martinez-Muriana A., Fattorelli N., Wolfs L. (2023). The γ-secretase substrate proteome and its role in cell signaling regulation. Mol. Cell.

[209] Lomize A.L., Schnitzer K.A., Todd S.C., Cherepanov S., Outeiral C., Deane C.M. (2022). Membranome 3.0: database of single-pass membrane proteins with AlphaFold models. Protein Sci..

[210] Zhou B., Lin W., Long Y., Yang Y., Zhang H., Wu K. (2022). Notch signaling pathway: architecture, disease, and therapeutics. Signal. Transduct. Target Ther..

[211] Bu G. (2009). Apolipoprotein E and its receptors in Alzheimer's disease: pathways, pathogenesis and therapy. Nat. Rev. Neurosci..

[212] Inoue E., Deguchi-Tawarada M., Togawa A., Matsui C., Arita K., Katahira-Tayama S. (2009). Synaptic activity prompts γ-secretase-mediated cleavage of EphA4 and dendritic spine formation. J. Cell Biol..

[213] Okamoto I., Kawano Y., Murakami D., Sasayama T., Araki N., Miki T. (2001). Proteolytic release of CD44 intracellular domain and its role in the CD44 signaling pathway. J. Cell Biol..

[214] Marambaud P., Shioi J., Serban G., Georgakopoulos A., Sarner S., Nagy V. (2002). A presenilin-1/γ-secretase cleavage releases the E-cadherin intracellular domain and regulates disassembly of adherens junctions. EMBO J..

[215] Saleh A.Z., Fang A.T., Arch A.E., Neupane D., El Fiky A., Krolewski J.J. (2004). Regulated proteolysis of the IFNaR2 subunit of the interferon-alpha receptor. Oncogene.

[216] Marambaud P., Wen P.H., Dutt A., Shioi J., Takashima A., Siman R. (2003). A CBP binding transcriptional repressor produced by the PS1/ε-cleavage of N-cadherin is inhibited by PS1 FAD mutations. Cell.

[217] Parent A.T., Barnes N.Y., Taniguchi Y., Thinakaran G., Sisodia S.S. (2005). Presenilin attenuates receptor-mediated signaling and synaptic function. J. Neurosci..

[218] Weng X., Maxwell-Warburton S., Hasib A., Ma L., Kang L. (2022). The membrane receptor CD44: novel insights into metabolism. Trends Endocrinol. Metab..

[219] Chhibber-Goel J., Coleman-Vaughan C., Agrawal V., Sawhney N., Hickey E., Powell J.C. (2016). γ-Secretase activity is required for regulated intramembrane proteolysis of tumor necrosis factor (TNF) receptor 1 and TNF-mediated pro-apoptotic signaling. J. Biol. Chem..

[220] Linggi B., Carpenter G. (2006). ErbB receptors: new insights on mechanisms and biology. Trends Cell Biol.

[221] Merilahti J.A.M., Elenius K. (2019). γ-Secretase-dependent signaling of receptor tyrosine kinases. Oncogene.

[222] Lemmon M.A., Schlessinger J. (2010). Cell signaling by receptor tyrosine kinases. Cell.

[223] Merilahti J.A.M., Ojala V.K., Knittle A.M., Pulliainen A.T., Elenius K. (2017). Genome-wide screen of γ-secretase-mediated intramembrane cleavage of receptor tyrosine kinases. Mol. Biol. Cell.

[224] Tomita T., Tanaka S., Morohashi Y., Iwatsubo T. (2006). Presenilin-dependent intramembrane cleavage of ephrin-B1. Mol. Neurodegener..

[225] Wunderlich P., Glebov K., Kemmerling N., Tien N.T., Neumann H., Walter J. (2013). Sequential proteolytic processing of the triggering receptor expressed on myeloid cells-2 (TREM2) protein by ectodomain shedding and γ-secretase-dependent intramembranous cleavage. J. Biol. Chem..

[226] Siebel C., Lendahl U. (2017). Notch signaling in development, tissue homeostasis, and disease. Physiol. Rev..

[227] Ho D.M., Artavanis-Tsakonas S., Louvi A. (2020). The Notch pathway in CNS homeostasis and neurodegeneration. Wiley Interdiscip. Rev. Dev. Biol..

[228] Müller U.C., Deller T., Korte M. (2017). Not just amyloid: physiological functions of the amyloid precursor protein family. Nat. Rev. Neurosci..

[229] Dunot J., Ribera A., Pousinha P.A., Marie H. (2023). Spatiotemporal insights of APP function. Curr. Opin. Neurobiol..

[230] Cavallaro U., Dejana E. (2011). Adhesion molecule signalling: not always a sticky business. Nat. Rev. Mol. Cell Biol..

[231] Boulton M.E., Cai J., Grant M.B. (2008). γ-Secretase: a multifaceted regulator of angiogenesis. J. Cell. Mol. Med..

[232] Biemesderfer D. (2006). Regulated intramembrane proteolysis of megalin: linking urinary protein and gene regulation in proximal tubule?. Kidney Int..

[233] Fornetti J., Flanders K.C., Henson P.M., Tan A.C., Borges V.F., Schedin P. (2016). Mammary epithelial cell phagocytosis downstream of TGF-β3 is characterized by adherens junction reorganization. Cell Death Differ..

[234] Restituito S., Khatri L., Ninan I., Mathews P.M., Liu X., Weinberg R.J. (2011). Synaptic autoregulation by metalloproteases and γ-secretase. J. Neurosci..

[235] Warren N.A., Voloudakis G., Yoon Y., Robakis N.K., Georgakopoulos A. (2018). The product of the γ-secretase processing of ephrinB2 regulates VE-cadherin complexes and angiogenesis. Cell. Mol. Life Sci..

[236] Schulz B., Pruessmeyer J., Maretzky T., Ludwig A., Blobel C.P., Saftig P. (2008). ADAM10 regulates endothelial permeability and T-Cell transmigration by proteolysis of vascular endothelial cadherin. Circ. Res..

[237] Bonn S., Seeburg P.H., Schwarz M.K. (2007). Combinatorial expression of α- and γ-protocadherins alters their presenilin-dependent processing. Mol. Cell Biol..

[238] Pancho A., Aerts T., Mitsogiannis M.D., Seuntjens E. (2020). Protocadherins at the crossroad of signaling pathways. Front. Mol. Neurosci..

[239] Kim J., Chang A., Dudak A., Federoff H.J., Lim S.T. (2011). Characterization of nectin processing mediated by presenilin-dependent γ-secretase. J. Neurochem..

[240] Saura C.A., Servian-Morilla E., Scholl F.G. (2011). Presenilin/γ-secretase regulates neurexin processing at synapses. PLoS One.

[241] Cornejo F., Cortes B.I., Findlay G.M., Cancino G.I. (2021). LAR receptor tyrosine phosphatase family in healthy and diseased brain. Front. Cell Dev. Biol..

[242] Nyborg A.C., Ladd T.B., Zwizinski C.W., Lah J.J., Golde T.E. (2006). Sortilin, SorCS1b, and SorLA Vps10p sorting receptors, are novel γ-secretase substrates. Mol. Neurodegener..

[243] Bronfman F.C. (2007). Metalloproteases and γ-secretase: new membrane partners regulating p75 neurotrophin receptor signaling?. J. Neurochem..

[244] Kim D.Y., Carey B.W., Wang H., Ingano L.A., Binshtok A.M., Wertz M.H. (2007). BACE1 regulates voltage-gated sodium channels and neuronal activity. Nat. Cell Biol..

[245] Sachse C.C., Kim Y.H., Agsten M., Huth T., Alzheimer C., Kovacs D.M. (2013). BACE1 and presenilin/γ-secretase regulate proteolytic processing of KCNE1 and 2, auxiliary subunits of voltage-gated potassium channels. FASEB J..

[246] Schulte A., Schulz B., Andrzejewski M.G., Hundhausen C., Mletzko S., Achilles J. (2007). Sequential processing of the transmembrane chemokines CX3CL1 and CXCL16 by α- and γ-secretases. Biochem. Biophys. Res. Commun..

[247] Elzinga B.M., Twomey C., Powell J.C., Harte F., McCarthy J.V. (2009). Interleukin-1 receptor type 1 is a substrate for γ-secretase-dependent regulated intramembrane proteolysis. J. Biol. Chem..

[248] Kuhn P.H., Marjaux E., Imhof A., De Strooper B., Haass C., Lichtenthaler S.F. (2007). Regulated intramembrane proteolysis of the interleukin-1 receptor II by α-, β-, and γ-secretase. J. Biol. Chem..

[249] Pasqualon T., Pruessmeyer J., Weidenfeld S., Babendreyer A., Groth E., Schumacher J. (2015). A transmembrane C-terminal fragment of syndecan-1 is generated by the metalloproteinase ADAM17 and promotes lung epithelial tumor cell migration and lung metastasis formation. Cell. Mol. Life Sci..

[250] Wang R., Tang P., Wang P., Boissy R.E., Zheng H. (2006). Regulation of tyrosinase trafficking and processing by presenilins: partial loss of function by familial Alzheimer's disease mutation. Proc. Natl. Acad. Sci. U. S. A..

[251] Ponta H., Sherman L., Herrlich P.A. (2003). CD44: from adhesion molecules to signalling regulators. Nat. Rev. Mol. Cell Biol..

[252] Schultz K., Grieger Lindner C., Li Y., Urbanek P., Ruschel A., Minnich K. (2018). Gamma secretase dependent release of the CD44 cytoplasmic tail upregulates IFI16 in *cd44-/-* tumor cells, MEFs and macrophages. PLoS One.

[253] Takahashi N., Knudson C.B., Thankamony S., Ariyoshi W., Mellor L., Im H.J. (2010). Induction of CD44 cleavage in articular chondrocytes. Arthritis Rheum..

[254] Lu Y., Wan J., Yang Z., Lei X., Niu Q., Jiang L. (2017). Regulated intramembrane proteolysis of the AXL receptor kinase generates an intracellular domain that localizes in the nucleus of cancer cells. FASEB J..

[255] Bae S.Y., Hong J.Y., Lee H.J., Park H.J., Lee S.K. (2015). Targeting the degradation of AXL receptor tyrosine kinase to overcome resistance in gefitinib-resistant non-small cell lung cancer. Oncotarget.

[256] De Falco V., Tamburrino A., Ventre S., Castellone M.D., Malek M., Manie S.N. (2012). CD44 proteolysis increases CREB phosphorylation and sustains proliferation of thyroid cancer cells. Cancer Res..

[257] Senbanjo L.T., AlJohani H., Majumdar S., Chellaiah M.A. (2019). Characterization of CD44 intracellular domain interaction with RUNX2 in PC3 human prostate cancer cells. Cell Commun. Signal..

[258] Passarella D., Ciampi S., Di Liberto V., Zuccarini M., Ronci M., Medoro A. (2022). Low-density lipoprotein receptor-related protein 8 at the crossroad between cancer and neurodegeneration. Int. J. Mol. Sci..

[259] Akter J., Takatori A., Islam M.S., Nakazawa A., Ozaki T., Nagase H. (2014). Intracellular fragment of NLRR3 (NLRR3-ICD) stimulates ATRA-dependent neuroblastoma differentiation. Biochem. Biophys. Res. Commun..

[260] Liu B., Wang L., Shen L.L., Shen M.Z., Guo X.D., Wang T. (2012). RNAi-mediated inhibition of presenilin 2 inhibits glioma cell growth and invasion and is involved in the regulation of Nrg1/ErbB signaling. Neuro. Oncol..

[261] Park C.S., Kim O.S., Yun S.M., Jo S.A., Jo I., Koh Y.H. (2008). Presenilin 1/γ-secretase is associated with cadmium-induced E-cadherin cleavage and COX-2 gene expression in T47D breast cancer cells. Toxicol. Sci..

[262] Nami B., Wang Z. (2024). A non-canonical p75HER2 signaling pathway underlying trastuzumab action and resistance in breast cancer. Cells.

[263] Leocadio D., Mitchell A., Winder S.J. (2016). γ-Secretase dependent nuclear targeting of dystroglycan. J. Cell. Biochem..

[264] Stoyanova T., Goldstein A.S., Cai H., Drake J.M., Huang J., Witte O.N. (2012). Regulated proteolysis of Trop2 drives epithelial hyperplasia and stem cell self-renewal via β-catenin signaling. Genes Dev..

[265] Barbaud A., Lascombe I., Pechery A., Arslan S., Kleinclauss F., Fauconnet S. (2024). GW501516-mediated targeting of tetraspanin 15 regulates ADAM10-dependent N-cadherin cleavage in invasive bladder cancer cells. Cells.

[266] Degnin C.R., Laederich M.B., Horton W.A. (2011). Ligand activation leads to regulated intramembrane proteolysis of fibroblast growth factor receptor 3. Mol. Biol. Cell.

[267] Walter J., Kemmerling N., Wunderlich P., Glebov K. (2017). γ-Secretase in microglia - implications for neurodegeneration and neuroinflammation. J. Neurochem..

[268] Sachse S.M., Lievens S., Ribeiro L.F., Dascenco D., Masschaele D., Horre K. (2019). Nuclear import of the DSCAM-cytoplasmic domain drives signaling capable of inhibiting synapse formation. EMBO J..

[269] Brackenbury W.J., Isom L.L. (2011). Na channel β subunits: overachievers of the ion channel family. Front. Pharmacol..

[270] Jay T.R., von Saucken V.E., Landreth G.E. (2017). TREM2 in neurodegenerative diseases. Mol. Neurodegener..

[271] Rajebhosale P., Jone A., Johnson K.R., Hofland R., Palarpalar C., Khan S. (2024). Neuregulin1 nuclear signaling influences adult neurogenesis and regulates a schizophrenia susceptibility gene network within the mouse dentate gyrus. J. Neurosci..

[272] Ndoj K., Meurs A., Papaioannou D., Bjune K., Zelcer N. (2025). The low-density lipoprotein receptor: emerging post-transcriptional regulatory mechanisms. Atherosclerosis.

[273] Yang Y., Wu Y., Zhang S., Song W. (2013). High glucose promotes Aβ production by inhibiting APP degradation. PLoS One.

[274] Krogh A., Larsson B., von Heijne G., Sonnhammer E.L. (2001). Predicting transmembrane protein topology with a hidden Markov model: application to complete genomes. J. Mol. Biol..

[275] Breimann S. (2025).

[276] Tousseyn T., Thathiah A., Jorissen E., Raemaekers T., Konietzko U., Reiss K. (2009). ADAM10, the rate-limiting protease of regulated intramembrane proteolysis of Notch and other proteins, is processed by ADAMS-9, ADAMS-15, and the γ-secretase. J. Biol. Chem..

[277] Nagara Y., Hagiyama M., Hatano N., Futai E., Suo S., Takaoka Y. (2012). Tumor suppressor cell adhesion molecule 1 (CADM1) is cleaved by a disintegrin and metalloprotease 10 (ADAM10) and subsequently cleaved by γ-secretase complex. Biochem. Biophys. Res. Commun..

[278] Maetzel D., Denzel S., Mack B., Canis M., Went P., Benk M. (2009). Nuclear signalling by tumour-associated antigen EpCAM. Nat. Cell Biol.

[279] Carey B.W., Kim D.Y., Kovacs D.M. (2007). Presenilin/γ-secretase and α-secretase-like peptidases cleave human MHC class I proteins. Biochem. J..

[280] Maretzky T., Schulte M., Ludwig A., Rose-John S., Blobel C., Hartmann D. (2005). L1 is sequentially processed by two differently activated metalloproteases and presenilin/γ-secretase and regulates neural cell adhesion, cell migration, and neurite outgrowth. Mol. Cell Biol..

[281] Anders L., Mertins P., Lammich S., Murgia M., Hartmann D., Saftig P. (2006). Furin-, ADAM 10-, and γ-secretase-mediated cleavage of a receptor tyrosine phosphatase and regulation of β-catenin's transcriptional activity. Mol. Cell Biol..

[282] Kim D.Y., Ingano L.A., Carey B.W., Pettingell W.H., Kovacs D.M. (2005). Presenilin/γ-secretase-mediated cleavage of the voltage-gated sodium channel β2-subunit regulates cell adhesion and migration. J. Biol. Chem..

[283] Six E., Ndiaye D., Laabi Y., Brou C., Gupta-Rossi N., Israel A. (2003). The Notch ligand Delta1 is sequentially cleaved by an ADAM protease and γ-secretase. Proc. Natl. Acad. Sci. U. S. A..

[284] Perrone C., Pomella S., Cassandri M., Pezzella M., Milano G.M., Colletti M. (2022). MET inhibition sensitizes rhabdomyosarcoma cells to NOTCH signaling suppression. Front. Oncol..

[285] Vevea J.D., Kusick G.F., Courtney K.C., Chen E., Watanabe S., Chapman E.R. (2021). Synaptotagmin 7 is targeted to the axonal plasma membrane through γ-secretase processing to promote synaptic vesicle docking in mouse hippocampal neurons. Elife.

[286] Jutras I., Laplante A., Boulais J., Brunet S., Thinakaran G., Desjardins M. (2005). γ-Secretase is a functional component of phagosomes. J. Biol. Chem..

[287] Lichtenthaler S.F., Tschirner S.K., Steiner H. (2022). Secretases in Alzheimer's disease: novel insights into proteolysis of APP and TREM2. Curr. Opin. Neurobiol..

[288] Na H.W., Shin W.S., Ludwig A., Lee S.T. (2012). The cytosolic domain of protein-tyrosine kinase 7 (PTK7), generated from sequential cleavage by a disintegrin and metalloprotease 17 (ADAM17) and γ-secretase, enhances cell proliferation and migration in colon cancer cells. J. Biol. Chem..

[289] Yurrita M.M., Fernandez-Munoz B., Del Castillo G., Martin-Villar E., Renart J., Quintanilla M. (2014). Podoplanin is a substrate of presenilin-1/γ-secretase. Int. J. Biochem. Cell Biol..

[290] Zou Z., Chung B., Nguyen T., Mentone S., Thomson B., Biemesderfer D. (2004). Linking receptor-mediated endocytosis and cell signaling: evidence for regulated intramembrane proteolysis of megalin in proximal tubule. J. Biol. Chem..

[291] Rudan Njavro J., Klotz J., Dislich B., Wanngren J., Shmueli M.D., Herber J. (2020). Mouse brain proteomics establishes MDGA1 and CACHD1 as in vivo substrates of the Alzheimer protease BACE1. FASEB J..

[292] Goldschneider D., Rama N., Guix C., Mehlen P. (2008). The neogenin intracellular domain regulates gene transcription via nuclear translocation. Mol. Cell Biol..

[293] Willem M. (2016). Proteolytic processing of Neuregulin-1. Brain Res. Bull..

[294] Kasuga K., Kaneko H., Nishizawa M., Onodera O., Ikeuchi T. (2007). Generation of intracellular domain of insulin receptor tyrosine kinase by γ-secretase. Biochem. Biophys. Res. Commun..

[295] Schulz J.G., Annaert W., Vandekerckhove J., Zimmermann P., De Strooper B., David G. (2003). Syndecan 3 intramembrane proteolysis is presenilin/γ-secretase-dependent and modulates cytosolic signaling. J. Biol. Chem..

[296] Cowan J.W., Wang X., Guan R., He K., Jiang J., Baumann G. (2005). Growth hormone receptor is a target for presenilin-dependent γ-secretase cleavage. J. Biol. Chem..

[297] Kanning K.C., Hudson M., Amieux P.S., Wiley J.C., Bothwell M., Schecterson L.C. (2003). Proteolytic processing of the p75 neurotrophin receptor and two homologs generates C-terminal fragments with signaling capability. J. Neurosci..

[298] Yong Y.L., Zhang R.Y., Liu Z.K., Wei D., Shang Y.K., Wu J. (2019). γ-Secretase complex-dependent intramembrane proteolysis of CD147 regulates the Notch1 signaling pathway in hepatocellular carcinoma. J. Pathol..

[299] Eiraku M., Tohgo A., Ono K., Kaneko M., Fujishima K., Hirano T. (2005). DNER acts as a neuron-specific notch ligand during Bergmann glial development. Nat. Neurosci..

[300] Blair C.R., Stone J.B., Wells R.G. (2011). The type III TGF-β receptor betaglycan transmembrane-cytoplasmic domain fragment is stable after ectodomain cleavage and is a substrate of the intramembrane protease γ-secretase. Biochim. Biophys. Acta.

[301] Zhou F., Gong K., Song B., Ma T., van Laar T., Gong Y. (2012). The APP intracellular domain (AICD) inhibits wnt signalling and promotes neurite outgrowth. Biochim. Biophys. Acta.

[302] Mi K., Johnson G.V. (2007). Regulated proteolytic processing of LRP6 results in release of its intracellular domain. J. Neurochem..

[303] Lyu J., Yamamoto V., Lu W. (2008). Cleavage of the Wnt receptor Ryk regulates neuronal differentiation during cortical neurogenesis. Dev. Cell.

[304] Kolliopoulos C., Ali M.M., Castillejo-Lopez C., Heldin C.H., Heldin P. (2022). CD44 depletion in glioblastoma cells suppresses growth and stemness and induces senescence. Cancers (Basel).

[305] Stoeck A., Shang L., Dempsey P.J. (2010). Sequential and γ-secretase-dependent processing of the betacellulin precursor generates a palmitoylated intracellular-domain fragment that inhibits cell growth. J. Cell Sci..

[306] Chen Z., Kapus A., Khatri I., Kos O., Zhu F., Gorczynski R.M. (2018). Cell membrane-bound CD200 signals both via an extracellular domain and following nuclear translocation of a cytoplasmic fragment. Leuk. Res..

[307] Bloch L., Sineshchekova O., Reichenbach D., Reiss K., Saftig P., Kuro-o M. (2009). Klotho is a substrate for α-, β- and γ-secretase. FEBS Lett..

[308] Kenchappa R.S., Zampieri N., Chao M.V., Barker P.A., Teng H.K., Hempstead B.L. (2006). Ligand-dependent cleavage of the P75 neurotrophin receptor is necessary for NRIF nuclear translocation and apoptosis in sympathetic neurons. Neuron.

[309] Marcel N., Sarin A. (2016). Notch1 regulated autophagy controls survival and suppressor activity of activated murine T-regulatory cells. Elife.

[310] Aleksander S.A., Balhoff J., Carbon S., Cherry J.M., Drabkin H.J., The Gene Ontology Consortium (2023). The Gene Ontology knowledgebase in 2023. Genetics.

[311] Limbourg A., Ploom M., Elligsen D., Sorensen I., Ziegelhoeffer T., Gossler A. (2007). Notch ligand Delta-like 1 is essential for postnatal arteriogenesis. Circ. Res..

[312] Marron M.B., Singh H., Tahir T.A., Kavumkal J., Kim H.Z., Koh G.Y. (2007). Regulated proteolytic processing of Tie1 modulates ligand responsiveness of the receptor-tyrosine kinase Tie2. J. Biol.Chem..

[313] Sutcliffe J.G., Hedlund P.B., Thomas E.A., Bloom F.E., Hilbush B.S. (2011). Peripheral reduction of β-amyloid is sufficient to reduce brain β-amyloid: implications for Alzheimer's disease. J. Neurosci. Res..

[314] Kim T.H., Shivdasani R.A. (2011). Notch signaling in stomach epithelial stem cell homeostasis. J. Exp. Med..

[315] Taniguchi Y., Kim S.H., Sisodia S.S. (2003). Presenilin-dependent "γ-secretase" processing of deleted in colorectal cancer (DCC). J. Biol. Chem..

[316] Galichet A., Weibel M., Heizmann C.W. (2008). Calcium-regulated intramembrane proteolysis of the RAGE receptor. Biochem. Biophys. Res. Commun..

[317] Yuasa T., Amo K., Ishikura S., Nagaya H., Uchiyama K., Hashida S. (2014). Development of in vitro model of insulin receptor cleavage induced by high glucose in HepG2 cells. Biochem. Biophys. Res. Commun..

[318] Schneider M., Allman A., Maillard I. (2023). Regulation of immune cell development, differentiation and function by stromal Notch ligands. Curr. Opin. Cell Biol..

[319] Pont M.J., Hill T., Cole G.O., Abbott J.J., Kelliher J., Salter A.I. (2019). γ-Secretase inhibition increases efficacy of BCMA-specific chimeric antigen receptor T cells in multiple myeloma. Blood.

[320] Liu C., Nikain C., Li Y.M. (2023). γ-Secretase fanning the fire of innate immunity. Biochem. Soc. Trans..

[321] Londino J.D., Gulick D., Isenberg J.S., Mallampalli R.K. (2015). Cleavage of signal regulatory protein α (SIRPα) enhances inflammatory signaling. J. Biol. Chem..

[322] Zurhove K., Nakajima C., Herz J., Bock H.H., May P. (2008). γ-Secretase limits the inflammatory response through the processing of LRP1. Sci. Signal..

[323] Mambole A., Baruch D., Nusbaum P., Bigot S., Suzuki M., Lesavre P. (2008). The cleavage of neutrophil leukosialin (CD43) by cathepsin G releases its extracellular domain and triggers its intramembrane proteolysis by presenilin/γ-secretase. J. Biol. Chem..

[324] Lehar S.M., Dooley J., Farr A.G., Bevan M.J. (2005). Notch ligands Delta1 and Jagged1 transmit distinct signals to T-cell precursors. Blood.

[325] Lahey K.C., Varsanyi C., Wang Z., Aquib A., Gadiyar V., Rodrigues A.A. (2024). Regulation of Mertk surface expression via ADAM17 and γ-secretase proteolytic processing. Int. J. Mol. Sci..

[326] Pan Y., Lin M.H., Tian X., Cheng H.T., Gridley T., Shen J. (2004). γ-Secretase functions through Notch signaling to maintain skin appendages but is not required for their patterning or initial morphogenesis. Dev. Cell.

[327] Watt B., van Niel G., Raposo G., Marks M.S. (2013). PMEL: a pigment cell-specific model for functional amyloid formation. Pigment Cell Melanoma Res..

[328] Alghamri M.S., Sharma P., Williamson T.L., Readler J.M., Yan R., Rider S.D. (2021). MAGI-1 PDZ2 domain blockade averts adenovirus infection via enhanced proteolysis of the apical coxsackievirus and adenovirus receptor. J. Virol..

[329] Foveau B., Ancot F., Leroy C., Petrelli A., Reiss K., Vingtdeux V. (2009). Down-regulation of the met receptor tyrosine kinase by presenilin-dependent regulated intramembrane proteolysis. Mol. Biol. Cell.

[330] Liu J., Fukunaga-Kalabis M., Li L., Herlyn M. (2014). Developmental pathways activated in melanocytes and melanoma. Arch. Biochem. Biophys..

[331] High F.A., Zhang M., Proweller A., Tu L., Parmacek M.S., Pear W.S. (2007). An essential role for Notch in neural crest during cardiovascular development and smooth muscle differentiation. J. Clin. Invest..

[332] Georgakopoulos A., Litterst C., Ghersi E., Baki L., Xu C., Serban G. (2006). Metalloproteinase/Presenilin1 processing of ephrinB regulates EphB-induced Src phosphorylation and signaling. EMBO J..

[333] Seki M., Watanabe A., Enomoto S., Kawamura T., Ito H., Kodama T. (2010). Human ROBO1 is cleaved by metalloproteinases and γ-secretase and migrates to the nucleus in cancer cells. FEBS Lett..

[334] Chow J.P., Fujikawa A., Shimizu H., Suzuki R., Noda M. (2008). Metalloproteinase- and γ-secretase-mediated cleavage of protein-tyrosine phosphatase receptor type Z. J. Biol. Chem..

[335] Czarnek M., Bereta J. (2020). Proteolytic processing of neuregulin 2. Mol. Neurobiol..

[336] Suzuki K., Hayashi Y., Nakahara S., Kumazaki H., Prox J., Horiuchi K. (2012). Activity-dependent proteolytic cleavage of neuroligin-1. Neuron.

[337] Liu S., Zhang Z., Li L., Yao L., Ma Z., Li J. (2023). ADAM10- and γ-secretase-dependent cleavage of the transmembrane protein PTPRT attenuates neurodegeneration in the mouse model of Alzheimer's disease. FASEB J..

[338] Lee H., Lee E.J., Song Y.S., Kim E. (2014). Long-term depression-inducing stimuli promote cleavage of the synaptic adhesion molecule NGL-3 through NMDA receptors, matrix metalloproteinases and presenilin/γ-secretase. Philos. Trans. R. Soc. Lond. B, Biol. Sci..

[339] Piao Y., Kimura A., Urano S., Saito Y., Taru H., Yamamoto T. (2013). Mechanism of intramembrane cleavage of alcadeins by γ-secretase. PLoS One.

[340] Pigoni M., Wanngren J., Kuhn P.H., Munro K.M., Gunnersen J.M., Takeshima H. (2016). Seizure protein 6 and its homolog seizure 6-like protein are physiological substrates of BACE1 in neurons. Mol. Neurodegener..

[341] Sakry D., Neitz A., Singh J., Frischknecht R., Marongiu D., Biname F. (2014). Oligodendrocyte precursor cells modulate the neuronal network by activity-dependent ectodomain cleavage of glial NG2. PLoS Biol..

[342] Wicher G., Larsson M., Fex Svenningsen A., Gyllencreutz E., Rask L., Aldskogius H. (2006). Low density lipoprotein receptor-related protein-2/megalin is expressed in oligodendrocytes in the mouse spinal cord white matter. J. Neurosci. Res..

[343] Fleck D., Garratt A.N., Haass C., Willem M. (2012). BACE1 dependent neuregulin processing: review. Curr. Alzheimer Res..

[344] Jabbehdari S., Oganov A.C., Rezagholi F., Mohammadi S., Harandi H., Yazdanpanah G. (2024). Age-related macular degeneration and neurodegenerative disorders: shared pathways in complex interactions. Surv. Ophthalmol..

[345] Uchida Y., Gomi F., Murayama S., Takahashi H. (2013). Calsyntenin-3 C-terminal fragment accumulates in dystrophic neurites surrounding Aβ plaques in tg2576 mouse and Alzheimer disease brains: its neurotoxic role in mediating dystrophic neurite formation. Am. J. Pathol..

[346] Schiffer D., Mellai M., Boldorini R., Bisogno I., Grifoni S., Corona C. (2018). The significance of chondroitin sulfate proteoglycan 4 (CSPG4) in human gliomas. Int. J. Mol. Sci..

[347] Schulte E.C., Fukumori A., Mollenhauer B., Hor H., Arzberger T., Perneczky R. (2015). Rare variants in β-Amyloid precursor protein (APP) and Parkinson's disease. Eur. J. Hum. Genet..

[348] McLeod J., Curtis N., Lewis H.D., Good M.A., Fagan M.J., Genever P.G. (2009). γ-Secretase-dependent cleavage of amyloid precursor protein regulates osteoblast behavior. FASEB J..

[349] Sobue Y., Takahashi N., Ohashi Y., Suzuki M., Nishiume T., Kobayakawa T. (2019). Inhibition of CD44 intracellular domain production suppresses bovine articular chondrocyte de-differentiation induced by excessive mechanical stress loading. Sci. Rep..

[350] Cheng H.T., Miner J.H., Lin M., Tansey M.G., Roth K., Kopan R. (2003). γ-Secretase activity is dispensable for mesenchyme-to-epithelium transition but required for podocyte and proximal tubule formation in developing mouse kidney. Development.

[351] Kaimori J.Y., Nagasawa Y., Menezes L.F., Garcia-Gonzalez M.A., Deng J., Imai E. (2007). Polyductin undergoes notch-like processing and regulated release from primary cilia. Hum. Mol. Genet..

